# Acceptor Engineering Produces Ultrafast Nonradiative Decay in NIR-II Aza-BODIPY Nanoparticles for Efficient Osteosarcoma Photothermal Therapy via Concurrent Apoptosis and Pyroptosis

**DOI:** 10.34133/research.0169

**Published:** 2023-06-19

**Authors:** Zhenxiong Shi, Hua Bai, Jiaxing Wu, Xiaofei Miao, Jia Gao, Xianning Xu, Yi Liu, Jiamin Jiang, Jiaqi Yang, Jiaxin Zhang, Tao Shao, Bo Peng, Huili Ma, Dan Zhu, Guojing Chen, Wenbo Hu, Lin Li, Wei Huang

**Affiliations:** ^1^ Frontiers Science Center for Flexible Electronics, Xi’an Institute of Flexible Electronics (IFE) and Xi’an Institute of Biomedical Materials & Engineering, Northwestern Polytechnical University, Xi’an 710072, China.; ^2^ Key Laboratory for Organic Electronics and Information Displays & Institute of Advanced Materials (IAM), Nanjing University of Posts & Telecommunications, Nanjing 210023, China.; ^3^ Key Laboratory of Flexible Electronics (KLOFE) and IAM, Nanjing Tech University, Nanjing 211800, China.; ^4^ Britton Chance Center for Biomedical Photonics-MoE Key Laboratory for Biomedical Photonics, Wuhan National Laboratory for Optoelectronics-Advanced Biomedical Imaging Facility, Huazhong University of Science and Technology, Wuhan 430074, China.; ^5^ Department of Orthopedics, Xijing Hospital, The Fourth Military Medical University, Xi’an 710032, China.; ^6^ The Institute of Flexible Electronics (IFE, Future Technologies), Xiamen University, Xiamen 361005, China.

## Abstract

Small-molecule photothermal agents (PTAs) with intense second near-infrared (NIR-II, 1,000 to 1,700 nm) absorption and high photothermal conversion efficiencies (PCEs) are promising candidates for treating deep-seated tumors such as osteosarcoma. To date, the development of small-molecule NIR-II PTAs has largely relied on fabricating donor–acceptor–donor (D–A–D/D′) structures and limited success has been achieved. Herein, through acceptor engineering, a donor–acceptor–acceptor (D–A–A′)-structured NIR-II aza-boron-dipyrromethene (aza-BODIPY) PTA (SW8) was readily developed for the 1,064-nm laser-mediated phototheranostic treatment of osteosarcoma. Changing the donor groups to acceptor groups produced remarkable red-shifts of absorption maximums from first near-infrared (NIR-I) regions (~808 nm) to NIR-II ones (~1,064 nm) for aza-BODIPYs (SW1 to SW8). Furthermore, SW8 self-assembled into nanoparticles (SW8@NPs) with intense NIR-II absorption and an ultrahigh PCE (75%, 1,064 nm). This ultrahigh PCE primarily originated from an additional nonradiative decay pathway, which showed a 100-fold enhanced decay rate compared to that shown by conventional pathways such as internal conversion and vibrational relaxation. Eventually, SW8@NPs performed highly efficient 1,064-nm laser-mediated NIR-II photothermal therapy of osteosarcoma *via* concurrent apoptosis and pyroptosis. This work not only illustrates a remote approach for treating deep-seated tumors with high spatiotemporal control but also provides a new strategy for building high-performance small-molecule NIR-II PTAs.

## Introduction

Osteosarcoma is the most common malignant bone tumor in children and adolescents, with a survival rate between 25% and 30% at 5 years [1]. The current standard therapy, which is surgical resection with pre- and post-operative chemotherapy, can result in severe operational injuries and drug-resistance concerns [2]. Photothermal therapy (PTT), which uses light to irradiate photothermal agents (PTAs) to generate local heat for tumor ablation, is an attractive option owing to its minimal invasiveness and negligible side effects [3]. Given the deep-seated feature of osteosarcoma [4–10], PTT activated by NIR-II light is of particular interest because the NIR-II light enables ultrahigh penetration depths that are unattainable by visible and near-infrared light [11–15]. However, the implementation of this NIR-II-mediated PTT in treating osteosarcoma has been largely hindered by the lack of suitable NIR-II PTAs. 

Small-molecule PTAs show considerable promise for clinical use because of their excellent biosafety and relatively high body clearance rates compared to inorganic and polymeric ones [7,16–20]. An ideal small-molecule PTA should satisfy two requirements: (a) intense NIR-II absorption to maximize the light energy harvest and (b) high photothermal conversion efficiency (PCE) for efficiently converting excitation energy into local heat [4,21,22]. However, few studies have been conducted on the design and development of efficient NIR-II-absorbing organic small-molecule PTAs [14,23–28]. Meanwhile, recent attempts for achieving small-molecule NIR-II PTAs primarily concentrated on D–A–D/D′-structured high-PCE organic small molecules prepared by donor engineering strategies and achieved limited successes [21]. New organic small-molecule NIR-II PTAs with desired properties are urgently required. In addition to donor segments, acceptor segments are equally important in determining the optical performance of D–A–D/D′ materials [29]. For example, the use of acceptor engineering strategies for improving optical properties in visible or near-infrared regions has been successful in organic photovoltaics [30]. Therefore, we speculated that acceptor engineering could produce ideal organic small-molecule NIR-II PTAs, which have been largely unexplored.

**Fig. 1.**
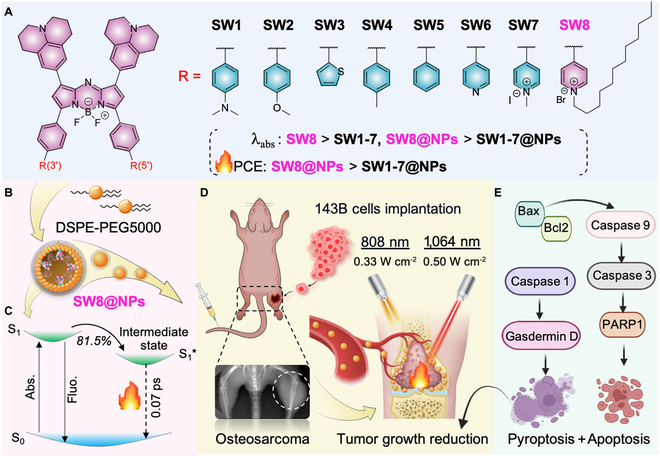
Rational design of novel aza-BODIPY-based NIR-II PTAs and its photothermal treatment for deep tumors. (A) Molecular structures of SW1 to SW8. (B) Preparation of SW8@NPs. (C) Kinetic mechanism proposed for SW8@NPs. (D) PTT effects of SW8@NPs for cellular and animal models of osteosarcoma under 808-/1,064-nm laser irradiation (created with http://BioRender.com). (E) Schematic illustrating molecular mechanisms underlying SW8@NPs-mediated anti-tumor effects under 808-/1,064-nm laser irradiation.

Generally, enhancing structure conjugation and increasing the flexibility of molecular motional fragments can yield high-performance NIR-II-absorbing small-molecule PTAs [31]. Our previous work presented the first example of NIR-II-emissive aza-BODIPY fluorophore (NJ1060) with a D–A–D′ structure, but its absorption maximum remained at approximately 808 nm [32]. Herein, using an acceptor engineering strategy, we explored small-molecule aza-BODIPY (SW8) and its nanoparticlization (SW8@NPs) to achieve intense NIR-II absorption and ultrahigh PCE for ablating osteosarcoma efficiently. The underlying mechanism was also studied. By changing donor groups at the 3- and 5-positions of aza-BODIPY cores into acceptor groups (Fig. 1A), we validated large red-shifts of absorption maximums from the NIR-I (~808 nm) region to NIR-II (~1,064 nm). Notably, nanoparticlization of SW8 (SW8@NPs, Fig. 1B) for practical biological applications showed an intense NIR-II absorption coefficient of 2,867 M^−1^ cm^−1^ at 1,064 nm and an ultrahigh PCE (75%) under laser irradiation, together assuring an exceptionally high NIR-II PTT effect. Furthermore, ultrafast spectroscopic studies ascribed this ultrahigh PCE to a nonradiative intermediate state. This dark intermediate depleted up to 80% of the excited population with a high decay rate of 1.3 × 10^13^ s^−1^ over conventional nonradiative decay channels such as internal conversion (Fig. 1C), resulting in the ultrahigh PCE [33,34]. Given these excellent properties, SW8@NPs could be used to realize a highly efficient in vivo PTT of osteosarcoma *via* concurrent apoptosis and pyroptosis (Fig. 1D and E), under low-intensity 1,064-nm laser (0.5 W cm^−2^, far below the clinical threshold of 1 W cm^−2^) irradiation. This study offers an alternative strategy for developing advanced organic small-molecule NIR-II materials for the diagnosis and treatment of deep-seated diseases.

## Results

**Fig. 2.**
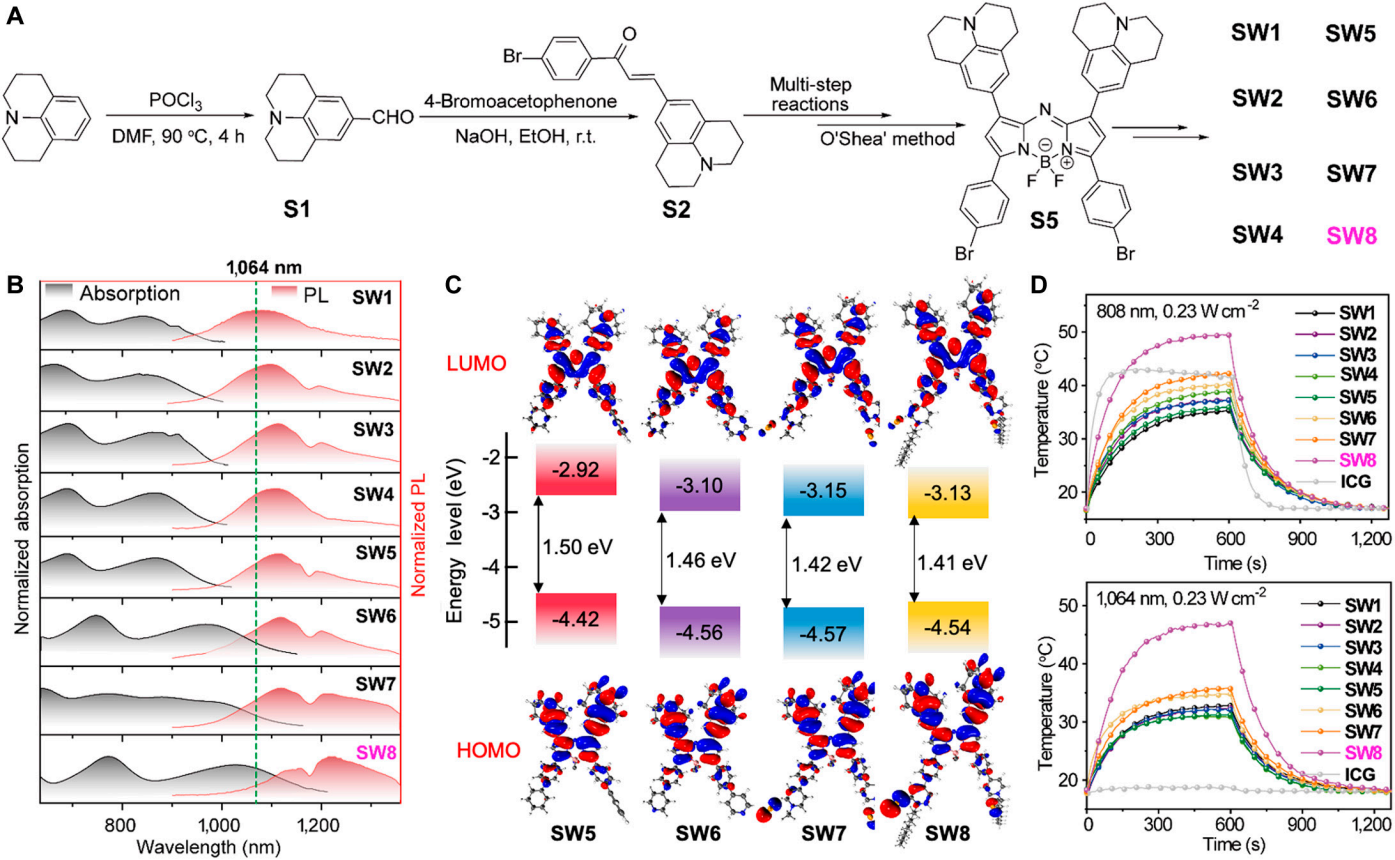
Synthesis and photophysical properties of SW1 to SW8. (A) Schematic diagram of SW1 to SW8 synthetic route. (B) Normalized absorption (black) and photoluminescence (PL) spectra (red) of SW1 to SW8 in dimethyl sulfoxide (DMSO). (C) Energy levels calculated for HOMO and LUMO. (D) Photothermal curves of SW1 to SW8 and ICG (4 × 10^−5^ mol l^−1^) in DMSO under laser (808/1,064 nm, 0.23 W cm^−2^) irradiation.

We synthesized a series of organic small-molecule PTAs (SW1 to SW8, Fig. 2A and Scheme S1) to validate the acceptor engineering strategy by incorporating various D′ or A′ segments at the 3- and 5-positions of aza-BODIPY [4,32,35]. The rotatable benzene ring is the π-conjugation linker between the aza-BODIPY core and the D′ or A′ segment. The alkyl chains were installed at the 3- and 5-positions of aza-BODIPY (SW8) for increased solubility and intermolecular interactions [36]. All compounds were characterized by ^1^H- and ^13^C-nuclear magnetic resonance (NMR) and matrix-assisted laser desorption/ionization time-of-flight mass spectrometry (MALDI-TOF) spectra (Supplementary Materials).

Figure 2B shows the normalized absorption (black) and photoluminescence (PL, red) spectra of SW1 to SW8, and the relevant data are summarized in Table S1. As expected, the absorption and PL spectra red-shifted with increasing degrees of electron deficiency for the group at the 3- or 5-position of aza-BODIPY (Figs. S1 and S2). Notably, remarkable red-shifts for the absorption and PL spectra occurred from SW5 to SW6, where the electron-donating benzene donor (D′) was changed to the pyridine acceptor (A′). Further increase in the electron deficiency of A′ caused more bathochromic wavelengths from SW6 to SW8, especially the red-shift of absorption wavelength is obvious, whereas the electron-donating moiety (from SW1 to SW5) exhibited no pronounced wavelength shift. In addition, the introduction of alkyl chains enhanced the *J*-aggregation of molecules, which is manifested by a red-shift of emission wavelength from SW6 to SW8. Time-dependent density functional theory (TD-DFT) calculations revealed a gradual decrease in the highest occupied molecular orbital (HOMO)–lowest unoccupied molecular orbital (LUMO) energy gap from SW5 to SW8 (Fig. 2C), consistent with the red-shifted spectra (Fig. 2B). Notably, the LUMO energy levels for SW5 to SW8 reduced in order, whereas the HOMO energy levels were almost unchanged. This phenomenon indicates that the acceptor segments at the 3- and 5-positions of aza-BODIPY significantly reduced the energy gaps, offering an alternative approach to construct new organic small-molecule NIR-II materials.

**Fig. 3.**
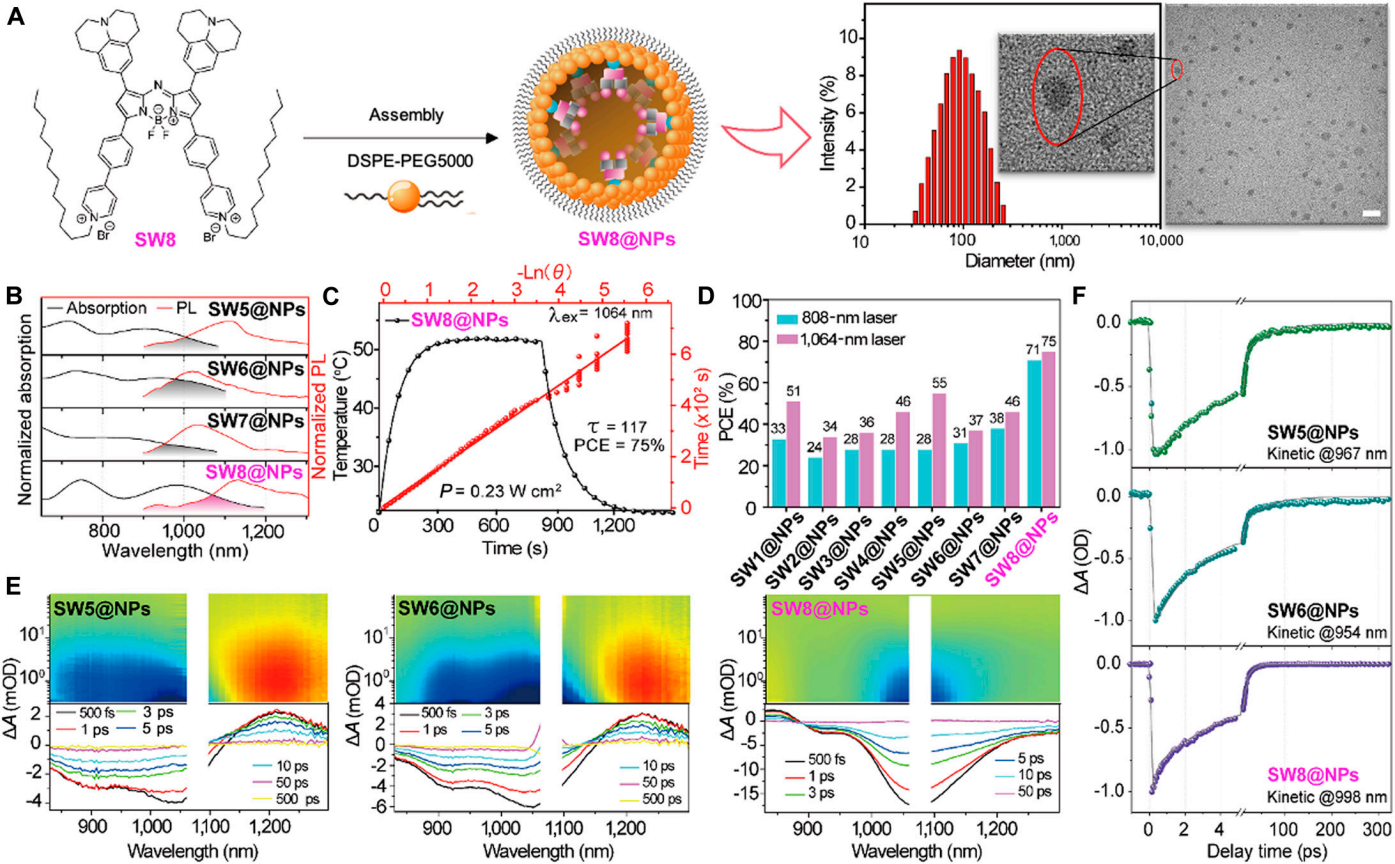
Preparation, characterization, and photophysical properties of SW1@NPs to SW8@NPs. (A) Preparation and characterization of SW8@NPs. Scale bar = 200 nm. (B) Normalized absorption (black) and PL (red) spectra of SW5@NPs to SW8@NPs in water. (C) Photothermal performance of SW8@NPs under 1,064-nm laser (0.2 mg ml^−1^, 0.23 W cm^−2^) irradiation by cooling to room temperature with linear analysis. (D) The PCEs of SW1@NPs to SW8@NPs under 808-/1,064-nm laser (0.2 mg ml^−1^, 0.23 W cm^−2^) irradiation. (E) Full contour femtosecond transient absorption (fs-TA) mappings and fs-TA plots at selected delay times. (F) Representative kinetic decay curves within ground state bleaching (GSB) regions and fitting lines.

We further demonstrated the intense NIR-II absorption of SW8 accompanied by moderate fluorescence at approximately 1,230 nm (Fig. S3). SW8 exhibited superior photothermal effects compared to SW1 to SW7 (Fig. 2D), affording remarkable potential for NIR-II-mediated PTT. Owing to these properties, **SW8** was encapsulated into an amphiphilic matrix to form water-soluble nanoparticles (SW8@NPs) through typical self-assembly nanoparticlization (left of Fig. 3A). Transmission electron microscopy (TEM) and dynamic light scattering (DLS) revealed uniformly spherical morphologies of SW1@NPs to SW8@NPs with a mean diameter of ~100 nm (right of Fig. 3A and Figs. S4 and S5). Because of the low absorption of SW1@NPs to SW4@NPs at 1,064 nm (Fig. S7), we explored the photophysical properties of SW5@NPs to SW8@NPs for potential phototheranostic applications mediated by 1,064-nm laser irradiation. Compared to SW5@NPs to SW7@NPs, SW8@NPs showed a more intense NIR-II absorption of 2,867 M^−1^ cm^−1^ at 1,064 nm and moderate NIR-II fluorescence (Fig. 3B), affording NIR-II fluorescence diagnostic functionality. The PCE of SW8@NPs under 1,064-nm laser irradiation was determined to be as high as 75% (Fig. 3C), which is a remarkable improvement compared to those of SW1@NPs to SW7@NPs (Fig. 3D and Figs. S8 to S10). Furthermore, we demonstrated the excellent photothermal stability of SW8@NPs under 1,064-nm laser irradiation (Fig. S9a). Taken together, owing to these properties, SW8@NPs are an excellent organic PTA for 1,064-nm laser-mediated in vivo phototheranostic applications.

To elucidate the underlying reason for the ultrahigh PCE, we probed the excited-state dynamics using fs-TA spectra. Typically, GSB with negative signals in fs-TA mapping reflects the nonradiative depletion of the excited population (top of Fig. 3E), thus deciphering which is the key to elucidating the mechanism of photothermal effects [37,38]. SW8@NPs showed more facile nonradiative decay than SW5/6@NPs, and SW6@NPs as evidenced by the fastest deactivation of the excited population within the GSB region (Fig. 3E). For more detailed information, we extracted the fs-TA plots (bottom of Fig. 3E) and representative kinetic curves (Fig. 3F) from fs-TA mapping. As shown in the fs-TA plots, the GSB signal of SW8@NPs decayed entirely at 50 ps, whereas those of SW5@NPs and SW6@NPs lived up to 500 ps, further validating the faster nonradiative decay for SW8@NPs, which is favorable for photothermal effects. Furthermore, the representative kinetic curve of SW8@NPs within the GSB region reveals three components with time constants of 0.077 (τ_1_), 3.11 (τ_2_), and 13.3 ps (τ_3_) (Table S2), all collectively contributing to photothermal effects. Notably, SW8@NPs exhibited a short-lived femtosecond component for the nonradiative decay with an unbeatable rate of 1.3 × 10^13^ s^−1^, whereas SW5/6@NPs exhibited two considerably long-lived components for nonradiative decay (Fig. 3E and Table S2). This femtosecond component for SW8@NPs affords an additional nonradiative decay pathway with an unbeatable rate to deplete up to 81.5% of the excited population back to the ground states (S_0_), which should be the primary reason for its superior photothermal effects. The sub-10-ps component was assigned to vibrational relaxation. The faster vibrational relaxation for SW6@NPs (2.5 ps vs. 5 ps for SW5@NPs and 3.1 ps for SW8@NPs) originated from the more substantial overlap between its absorption and PL spectra, as outlined in Fig. 3B. The component ranging from 10 to 100 ps was attributed to the conventional internal conversion from S_1_ to S_0_. The accelerated S_1_ → S_0_ internal conversion for SW5@NPs (56 ps), compared to SW6@NPs (43 ps) and SW8@NPs (13.3 ps), originated from the red-shifted absorption, namely, the narrow band gap. This was because the nonradiative coupling probability between the low vibrational levels of S_1_ and high vibrational levels of S_0_ became dominated with the reduced band gap, as stated by the energy gap rule. Considering the above results, it is apparent that the vibrational relaxation dominated the nonradiative decay for SW5@NPs and SW6@NPs. The higher vibrational relaxation rate of SW6@NPs (4 × 10^11^ s^−1^), compared to that of SW5@NPs (2 × 10^11^ s^−1^), improved its photothermal effects (Fig. 3E and Table S2). In the case of SW8@NPs, the most competitive nonradiative decay pathway with an approximately 100-fold accelerated rate of 1.3 × 10^13^ s^−1^ readily beat the conventional vibrational relaxation and internal conversion, thus dominating the nonradiative decay process for generating the ultrahigh PCE [37]. The origin of this ultrafast decay pathway was probably the intermolecular intermediate facilitated by the inter-side chain interaction (Fig. 1C).

**Fig. 4.**
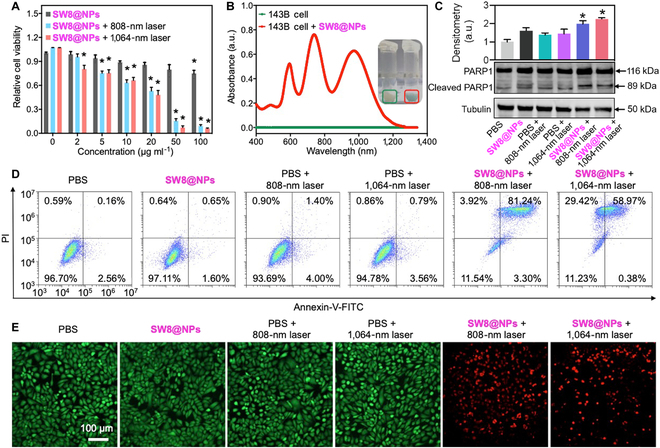
Photothermal effect of SW8@NPs under laser irradiation in vitro. (A) Viabilities of 143B cells at diverse concentrations of SW8@NPs irradiated by 808-/1,064-nm lasers (0.23 W cm^−2^) (*n* = 6, *P < 0.05 vs. SW8@NPs). (B) Cellular uptake of SW8@NPs to 143B cells. (C) Representative images (down) and quantification (up) of Western blots against PARP1, cleaved-PARP1, and tubulin after various treatment (*n* = 3, *P < 0.05 vs. PBS). (D) Cell apoptosis of 143B cells examined by flow cytometry. (E) Fluorescence imaging of 143B cells (96-well plates) stained with calcein AM and ethidium homodimer-1 (green: live cells, red: dead cells). Scale bar = 100 μm.

We first validated NIR-II activation of SW8@NPs in 7 different cell lines, namely, 143B (human-derived osteosarcoma cells), HepG2 (human hepatic carcinoma cells), L02 (human normal hepatocytes), Hela (human cervix carcinoma cells), hCMEC/D3 (human cardiac microvascular endothelial cells), HUVECs (human umbilical vein endothelial cells), and U87 (human astroglioma cells). As illustrated in Fig. 4A and Fig. S11, the cell viability exceeded 80% even at a high concentration of SW8@NPs (100 mg ml^−1^), clearly suggesting its adequate biocompatibility with cancer or normal cell lines. By contrast, upon irradiation with a 0.23 W cm^−2^ 808- or 1,064-nm laser for 5 min, a dose-dependent decline in cell viability was observed and reached 5.6% at 100 μg ml^−1^ SW8@NPs for the 143B cells. These results indicated that laser irradiation at 808 and 1,064 nm effectively induced photothermal effects of SW8@NPs in the cells. It is essential for nanoagents to be taken up efficiently by cells to achieve imaging and therapeutic performance [39]. In this study, SW8@NPs were investigated for their cellular uptake by the 143B cells. After incubating the nanoparticles and controls in the 143B cells for 4 h, strong NIR absorbance spectra were acquired from the SW8@NPs-treated group (Fig. 4B). This showed the effective incorporation of SW8@NPs into 143B cells, which supported that phototoxicity was not due to temperature increases in culture mediums and provided a basis for exploiting mechanisms underlying lethality effects induced by phagocytized SW8@NPs.

We studied SW8@NPs-induced cell death pathways under irradiation further. The cleavage of poly (adenosine diphosphate-ribose) polymerase-1 (PARP1) promotes apoptosis by preventing DNA repair, which is a hallmark of early apoptosis [40]. As shown in Fig. 4C, SW8@NPs with laser (808 or 1,064 nm) irradiation significantly upregulated the expression of cleaved-PARP1 for the 143B cells, compared with the vehicle control. Moreover, apoptosis of 143B cells was quantified by flow cytometry. As depicted in Fig. 4D, the apoptosis results were shown in a four-quadrant diagram: dots in the upper left quadrant (Q1) represented dead cells; dots in the lower right quadrant represented late apoptotic cells (Q2); dots in the lower left (Q3) quadrant represented living cells; and dots in the upper right represented early apoptotic cells (Q4). The sum of Q2 and Q4 represented the total cell apoptosis rate. The results showed that the apoptosis rate of the SW8@NPs + 808-nm laser group (84.54%) was higher than that of the SW8@NPs + 1,064-nm laser group (59.35%). However, when the total rate of cell death (Q1+Q2+Q4) was evaluated, no significant difference was observed in the two groups. This was consistent with the live/dead results in Fig. 4E and indicated that SW8@NPs + 1,064-nm laser induces cell death by a mechanism other than apoptosis. These results were highly consistent with the expression of cleaved-PARP1 above, indicating that the SW8@NPs-induced PTT resulted in programmed cell death [41]. Finally, cell viability was visualized using calcein AM (green) and ethidium homodimer-1 (red) dyes. Live cells are green and dead cells are red [42]. Similar to the flow cytometry results, the apparent red fluorescence for the “SW8@NPs + 808- or 1,064-nm laser” group revealed a drastic increase in dead cells (Fig. 4E). However, phosphate buffered saline (PBS), SW8@NPs, and PBS + laser groups showed only green fluorescence, indicating that strong cytotoxicity was induced by the coexistence of SW8@NPs + laser irradiation. These data affirmed multifaceted in vitro anti-tumor effects of SW8@NPs under laser (808 or 1,064 nm) irradiation.

**Fig. 5.**
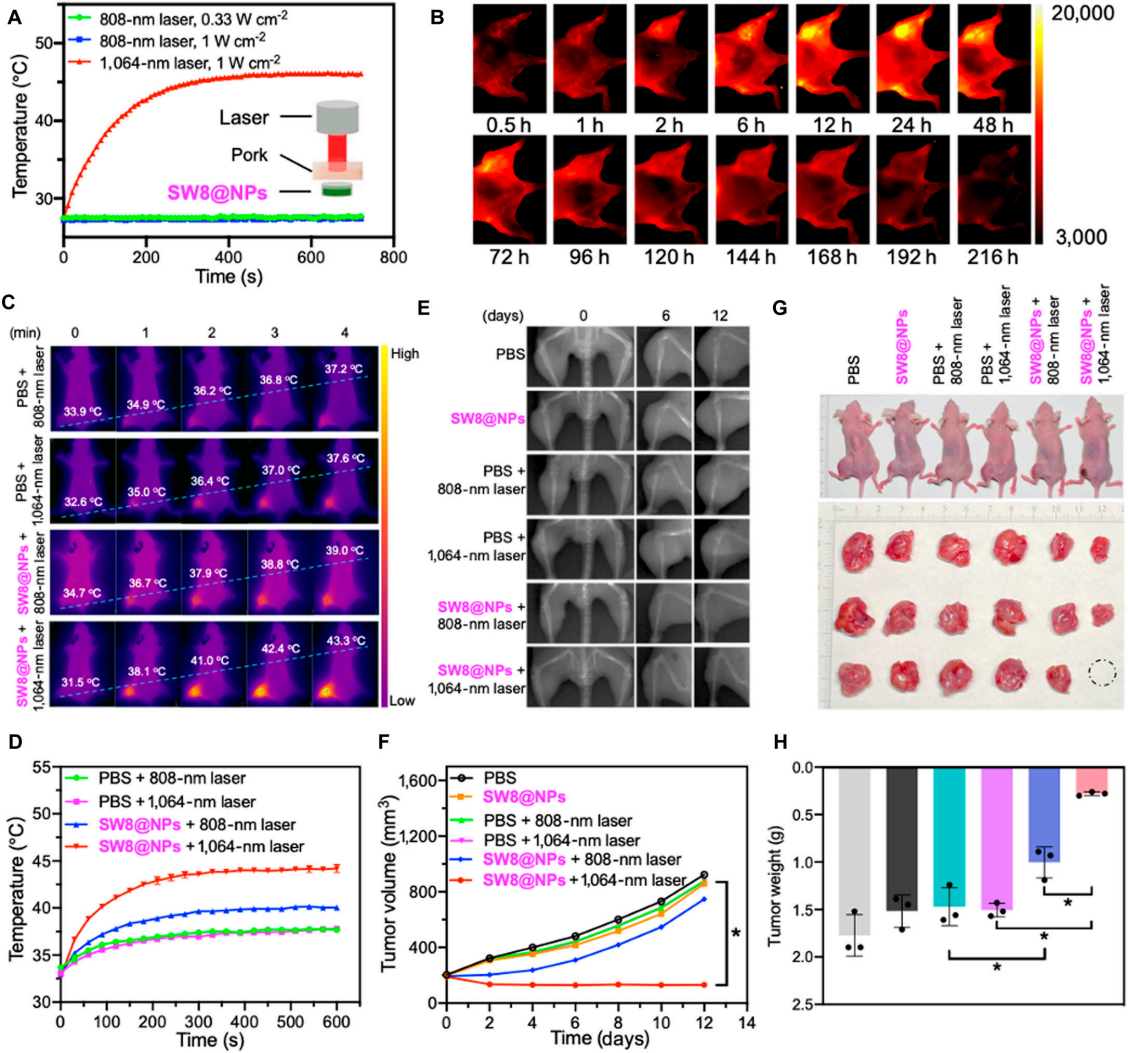
Photothermal effect of SW8@NPs under 808-/1,064-nm laser irradiation in vivo and its PTT of deep tumors. (A) One hundred microliters of SW8@NPs solution in a tube was covered with a piece of 15-mm-thick pork tissue, and temperature changes of SW8@NPs were recorded under laser (808/1,064 nm) irradiation. (B) Fluorescence images of 143B tumor-bearing mice (808-nm excitation, 1,000-nm-long pass, 0.27 W cm^−2^, 200 ms). (C) Photothermal images of 143B tumor-bearing mice irradiated by laser (808 nm = 0.33 W cm^−2^, 1,064 nm = 0.5 W cm^−2^). (D) Temperature profiles of tumor sites as functions of irradiation time (*n* = 3, *P < 0.05 vs. PBS + 1,064-nm laser). (E) X-ray images (60 kV, 20 ms) of mice at days 0, 6, and 12 after treatment. (F) Growth curves of in vivo tumor volumes. (G) High-definition photographs of mice and photographs of tumors removed on treatment day 12. The dashed circle indicated invisible tumors. (H) Weights of removed tumors (*n* = 3, *P < 0.05).

Encouraged by the PTT effect of SW8@NPs in vitro, we further evaluated the NIR-II PTT potential of SW8@NPs for treating orthotopic 143B tumor-bearing BALB/c nude mice. We first evaluated the stability of SW8@NPs in PBS. As shown in Fig. S12, the diameter of SW8@NPs did not change after 2 weeks of storage at 4 °C in PBS. We then compared the penetration depths of NIR-II (1,064 nm) and NIR-I (808 nm) lasers for SW8@NPs (Fig. 5A). Briefly, SW8@NPs solutions (1 mg ml^−1^) were covered with pork tissues of 15 mm thickness, which is sufficiently deep for mouse experiments. The maximum safe power density limit for the 808-nm laser is 0.33 W cm^−2^, and the maximum safe power density limit for the 1,064-nm laser is 1 W cm^−2^ [43]. In addition, the penetration experiment with pork fat showed that the penetration depth of the 808-nm laser was shallow, and increasing the laser power at 808 nm was of little significance for deep tumor treatment. The power densities of 0.5 W cm^−2^ (1,064-nm laser) are designed to ensure that the temperature of PTT is around 45 °C, thus reducing the side effects of PTT. According to this study, NIR-II (1,064 nm) phototherapy with SW8@NPs penetrated tissues better than NIR-I (808 nm). In order to evaluate the tumor-targeting ability of SW8@NPs, a mouse model of osteosarcoma was established by injecting 143B cells into the bone marrow cavity of the left tibia [15]. The tumor was allowed to reach a volume of approximately 80 to 100 mm^3^. The fluorescence signals of SW8@NPs (100 μl, 1 mg ml^−1^ by tail vein injection) were collected at an excitation wavelength of 808 nm. As shown in Fig. 5B, the fluorescence intensity gradually increased at the tumor site after injection and reached its peak level at approximately 12 to 24 h. Then, intensity of fluorescence decreased but remained strong till 72 h post-injection, illustrating the excellent retention of SW8@NPs in the tumor. Further, the tumor tissue and vital organs (heart, spleen, kidney, liver, and lung) of mice were harvested and imaged at designated time points (0.5 to 216 h) following injection. Figure S13 shows that SW8@NPs accumulated in the liver, spleen, and tumor over time and then decreased 72 h after injection. Moreover, SW8@NPs were gradually excreted through feces within 18 days (Fig. S14), indicating a hepatobiliary clearance pathway [44].

SW8@NPs were then tested in vivo for their ability to perform PTT in orthotopic 143B tumor-bearing mice. Continuous irradiation of tumor regions for 10 min using an 808-nm (0.33 W cm^−2^) or 1,064-nm laser (0.5 W cm^−2^) was performed 24 h after SW8@NPs injection and photothermal images were taken. As shown in Fig. 5C and D, the tumor surface temperature of the SW8@NPs-treated mouse rapidly reached 43.3 °C in 4 min after 1,064-nm laser irradiation, which is sufficient for killing the osteosarcoma tissue. By contrast, the tumor temperature increased slightly from ~33 to ~39 °C in the “SW8@NPs + 808-nm laser” group and was stable at ~37 °C in the “PBS + 808-/1,064-nm laser” groups. Based on these results, SW8@NPs exhibited excellent photothermal conversion capacity under 1,064-nm laser irradiation. This background helped us formulate a solid foundation for PTT activation by NIR-II in vivo.

Motivated by the tumor accumulation and excellent PCE of SW8@NPs, we sought to demonstrate its anti-tumor ability. X-ray imaging was used to monitor orthotopic tumor growth (Fig. 5E). The osteosarcoma mice were divided into six groups at random (*n* = 3): PBS, SW8@NPs, PBS + 808-nm laser, PBS + 1,064-nm laser, SW8@NPs + 808-nm laser, and SW8@NPs + 1,064-nm laser. At 24 h after SW8@NPs injections, the tumors were irradiated with laser for 10 min and repeated every other day for 12 days. The curve of the volume tendency for the “SW8@NPs +1,064-nm laser” group demonstrated complete tumor eradication during the 12 days of monitoring (Fig. 5F). By contrast, the other five treatments failed to suppress tumor growth, with an average increase in tumor volume of 4- to 5-fold. The weights of the dissected orthotopic tumors were also highly consistent at the end of the treatment (Fig. 5G and H). A significant weight loss was not observed in any group, either (Fig. S15). In summary, due to the deep-tissue penetration ability and high photothermal conversion efficiency, SW8@NPs irradiated by the 1,064-nm laser exhibited excellent anti-tumor efficacy in deep tissues.

**Fig. 6.**
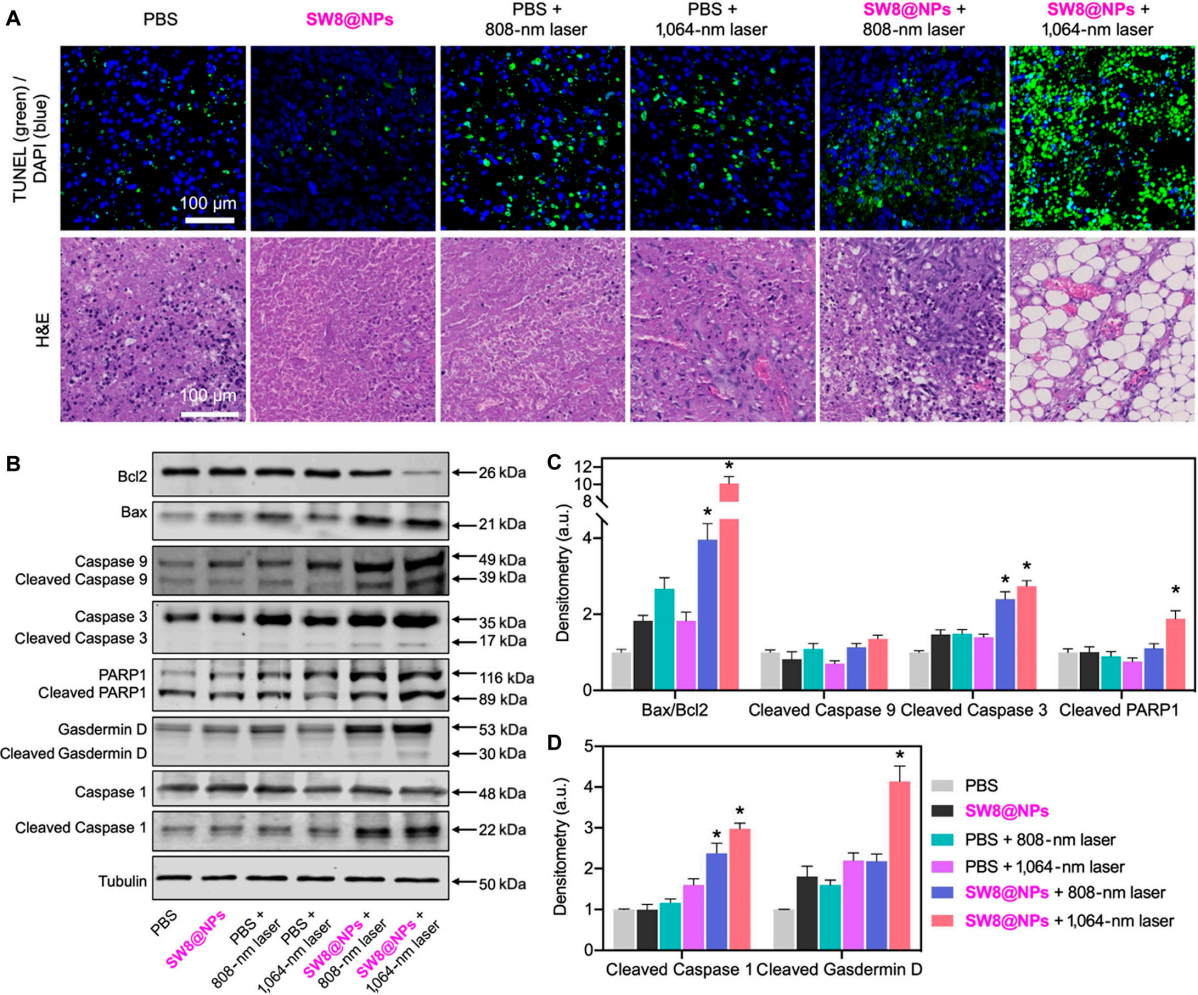
H&E and TUNEL staining of tumor tissues of mice and the anti-tumor mechanism of SW8@NPs with laser irradiation. (A) H&E and TUNEL staining of tumor tissues of mice from different groups. (B) Representative Western blots of apoptosis- and pyroptosis-related proteins. (C) Normalized quantifications of apoptosis-related proteins in (B). (D) Normalized quantifications of pyroptosis-related proteins in (B) (*n* = 3, *P < 0.05 vs. PBS).

Furthermore, histology and immunohistochemical assays were conducted on the orthotopic tumors from different groups. As shown in Fig. 6A, much more apoptotic cells with green fluorescence were observed for the “SW8@NPs + 1,064-nm laser” group compared with the other five groups. Moreover, the resected tumor tissues were clearly destroyed in the “SW8@NPs + 1,064-nm laser” group, whereas the tumor tissues in other groups retained regular cell shapes with intact nuclei. A histological examination of the vital organs revealed no obvious edema damage or necrosis in all groups (Fig. S16). These data demonstrated that SW8@NPs-mediated NIR-II PTT is effective in treating deep-tissue tumors with a high degree of biosafety.

Finally, we investigated the anti-tumor mechanism of SW8@NPs with irradiation. According to previous studies and our in vitro data, PTT could lead to cell death *via* apoptosis pathways [45]. Therefore, we further explored whether the apoptosis pathways also existed for SW8@NPs-treated tumor tissues. Western blotting was used to analyze apoptosis-associated proteins (Bax, Bcl2, Caspase 9, Caspase 3, and PARP1). Increased Bax/Bcl2 ratios induce apoptosis by activating Caspase 9 and Caspase 3[46]. Cell apoptosis leads to the emergence of structurally damaged DNA fragments, thus activating PARP1 for repairing the structurally damaged DNA fragments [40]. As shown in Fig. 6**B** and C, SW8@NPs groups with irradiation showed significantly increased Bax/Bcl2 ratios as compared to the other groups. Subsequently, the increase in the Bax/Bcl2 ratio resulted in the enhanced expressions of cleaved Caspase 3 and PARP1. In addition to the apoptosis induction pathway, photo-activated pyroptosis induction may be an alternative therapeutic strategy against tumors. Pyroptosis is an immunogenic mode of programmed cell death [47]. Cell pyroptosis mainly relies on inflammasomes for activating parts of Caspase proteins (the Caspase-1 pathway is the classic pathway of cell pyroptosis) to cut Gasdermin proteins, and the activated Gasdermin proteins are translocated to membranes, forming holes, cell swellings, and cytoplasmic outflow and leading to cell membrane rupture. As shown in Fig. 6B and D, SW8@NPs significantly increased the levels of cleaved Caspase 1 and Gasdermin D under laser (808 or 1,064 nm) irradiation. Together, our results revealed, for the first time, that the SW8@NPs-mediated NIR-II PTT exerted anti-tumor effects mainly by stimulating concurrent apoptosis and pyroptosis.

## Conclusion

In summary, we reported on the design of a novel organic small-molecule PTA (SW8) and self-accessibility nanoparticles (SW8@NPs) with a high PCE (75%) in the NIR-II window (1,064 nm). Molecular excited-state dynamics analysis showed that this ultrahigh PCE primarily originated from an additional nonradiative decay pathway. A series of in vitro and in vivo experiments demonstrated for the first time that superior NIR-II PTT could effectively induce concurrent apoptosis and pyroptosis in osteosarcoma tissues. X-ray imaging revealed that deep-seated osteosarcoma could be completely ablated by precise 1,064-nm laser irradiation using SW8@NPs with minor side effects. It is our belief that design of organic small-molecule PTAs based on rational principles in the NIR-II window will benefit the practical clinical applications of photothermal activations and treatments in the future.

## Materials and Methods

All chemicals were purchased from Sigma-Aldrich or TCI. The ^1^H- and ^13^C-NMR spectra were obtained from a Bruker Ultra Shield Plus 500 MHz NMR instrument at 298 K by using CDCl_3_ or (CD_3_)_2_SO as the solvent. Mass spectra were recorded by MALDI-TOF (Bruker, AutoFlex III system). NPs are prepared using an ultrasonic crusher (VCX-130), and NP size was obtained from DLS on Zetasizer Nanoseries (Zetasizer nano ZS, UK). Morphology and size of NPs were determined using HT7800 TEM. The UV–Visible–NIR absorption spectra were acquired using a Hitachi UH5700-spectrophotometer. The fluorescence emission was acquired via a steady-transient fluorescence spectrometer (FLS1000, Edinburgh Instruments, UK). Photothermal imaging was acquired for FLIR E40. The ultrafast spectroscopic test method can be referred to Ref.[48]. A flow sight imaging flow cytometer (CytoFLEX S) was used for flow cytometry experiments. Cell viability was tested by a multi-functional microporous detector (TECAN SPARK). NIR-II imaging was performed on an In-Vivo NIR-II imaging system (CRED2, France). Western blot was performed by using the LI-COR Odyssey Infrared Imaging System. Antibody information is summarized in Table S3. X-ray imaging of the mice was obtained by MODEL JYF-10D DENTAL X-RAY UNIT (Shenzhen Youlan Medical Health Co., Ltd, China). X-ray tube: XD2-1.4/85, and its power output is 60 kV.

### Theoretical calculations

All the calculations were based on DFT with the O3LYP functional and def2-svp basis set. All these calculations were performed with Gaussian 16.

### The cellular uptake of 143B cells treated with SW8@NPs

143B cells in the dish were incubated with 400 μg ml^−1^ SW8@NPs for 4 h, and then cells were harvested and sonicated for 5 min by adding EA with an ultrasonic disruptor. After centrifugation, the supernatant was collected and dried. The NIR absorption spectra of the sample was tested after adding 1 ml of PBS.

### Apoptosis assay

Apoptosis was measured with an annexin V-FITC apoptosis detection kit (Beyotime, China) depending on the manual.

### Live/dead staining

143B cells in 96-well plates were incubated with 50 μg ml^−1^ SW8@NPs for 4 h and then illuminated with laser (5 min). After incubation for an additional 4 h, 143B cells were washed 3 times with PBS and double staining with Calcein-AM and EthD-1 (Invitrogen) was used to detect live and dead cells. Cells were imaged with fluorescence microscopy (EVOS FL Auto 2).

### Western blotting

Western blotting was performed as described in our previous study [49].

### Laser penetration ability experiments

To evaluate the tissue penetration ability of the laser, 100 μl of the SW8@NPs solution (1 mg ml^−1^) in the tube was covered with a piece of 15-mm-thick pork tissue. Temperature changes of SW8@NPs (100 μl, 1 mg ml^−1^) were recorded under 808-nm (0.33 W cm^−2^ or 1 W cm^−2^) and 1,064-nm (1 W cm^−2^) irradiation, respectively.

### Photothermal anti-tumor therapy

In order to assess the anti-tumor efficacy of SW8@NPs, 18 mice were randomly divided into six groups. After various treatments, the body weights and tumor growth were monitored every other day. The tumors were collected and weighed at 12 days after treatments.

### Radiographic analysis

Digital x-ray images of all 18 mice were collected for 20 ms at 60 kV every other day for 12 days.

### Histology stain

Tissues were fixed in paraformaldehyde solution (4%) and stained using hematoxylin and eosin (H&E). To further assess the anti-tumor efficacy in the different groups, terminal deoxynucleotidyl transferase mediated dUTP nick-end labeling (TUNEL) in situ apoptosis detection was performed on tumor tissues (Promega Dead-End Colorimetric TUNEL system) using immunofluorescence.

## Data Availability

Data supporting the findings of this study are available in the main text or the Supplementary Materials.

## Supplementary Materials

Supplementary 1 Scheme S1. Synthetic route of NIR-II Aza-BODIPY photothermal agents (SW1 to SW8). Figs. S1 to S43 Tables. S1 to S4
