# Supplementary material for: Acceptor Engineering Produces Ultrafast Nonradiative Decay in NIR-II Aza-BODIPY Nanoparticles for Efficient Osteosarcoma Photothermal Therapy via Concurrent Apoptosis and Pyroptosis
Source: Research (Wash D C). 2023 Jun 19;6:0169. doi: 10.34133/research.0169 (PMC10278946; doi:10.34133/research.0169)
Supplement: Supplementary 1 — Scheme S1. Synthetic route of NIR-II Aza-BODIPY photothermal agents (SW1 to SW8). Figs. S1 to S43 Tables. S1 to S4 [file research.0169.f1.pdf]

Supporting Information

Acceptor Engineering Produces Ultrafast Nonradiative Decay in NIR-II Aza-BODIPY Nanoparticles for Efficient Osteosarcoma Photothermal Therapy via Concurrent Apoptosis and Pyroptosis

Zhenxiong Shi[1,&], Hua Bai[1,&], Jiaxing Wu[1], Xiaofei Miao[2], Jia Gao[5], Xianning Xu[1], Yi Liu[5], Jiamin Jiang[1], Jiaqi Yang[1], Jiaxin Zhang[1], Tao Shao[1], Bo Peng[1], Huili Ma[5], Dan Zhu[3], Guojing Chen[4], Wenbo Hu[1,*], Lin Li[1,5,6,*], Wei Huang[1,2,5,6,*]

[1]*Frontiers Science Center for Flexible Electronics, Xi'an Institute of Flexible Electronics (IFE) and Xi'an Institute of Biomedical Materials & Engineering, Northwestern Polytechnical University, Xi'an 710072, China.*

[2]*Key Laboratory for Organic Electronics and Information Displays & Institute of Advanced Materials (IAM), Nanjing University of Posts & Telecommunications, Nanjing 210023, China.*

[3]*Britton Chance Center for Biomedical Photonics-MoE Key Laboratory for Biomedical Photonics, Wuhan National Laboratory for Optoelectronics-Advanced Biomedical Imaging Facility, Huazhong University of Science and Technology, Wuhan, Hubei 430074, China.*

[4]*Department of Orthopedics, Xijing Hospital, Air Force Medical University of PLA, Xi'An, China.*

[5]*Key Laboratory of Flexible Electronics (KLOFE) & Institute of Advanced Materials (IAM), Nanjing Tech University (NanjingTech), Nanjing, 211800, China.*

[6]*The Institute of Flexible Electronics (IFE, Future Technologies), Xiamen University, Xiamen 361005, Fujian, China.*

[&]*Zhenxiong Shi and Hua Bai contributed equally to this work.*

*Address correspondence to: iamlli@nwpu.edu.cn, iamwbhu@nwpu.edu.cn, and vc@nwpu.edu.cn.

1. Synthesis and characterization

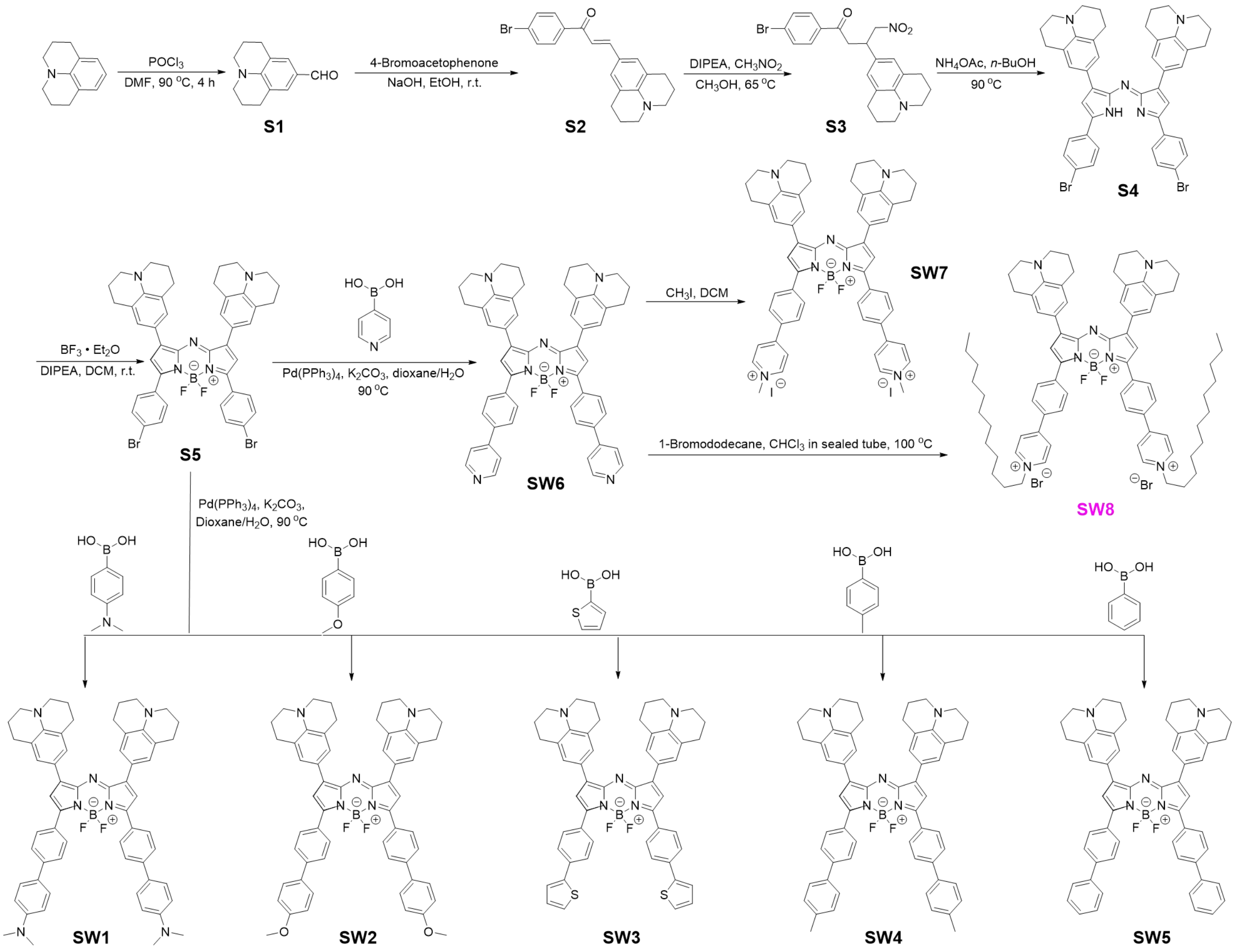

Scheme S1. Synthetic route of NIR-II Aza-BODIPY photothermal agents (**SW1-8**).

Preparation of compound S1

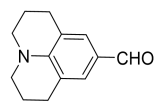

S1

Phosphorus oxychloride (1.17 g, 7.62 mmol) was slowly added dropwise into a 100 mL round bottom flask containing dry DMF (1.67 g, 22.87 mmol) at ice-salt bath conditions. Under the protection of N_2 atmosphere, the solution was stirred for another 2 h, and then obtained Vilsmeier-Haack reagent. Jilonidine (1.20 g, 6.93 mmol) in dry DMF (10 mL) was slowly added into the flask containing Vilsmeier-Haack reagent. After that, the solution was stirred at 90 °C for 4 h. Upon being cooled to room temperature, the mixture was poured into ice-water (100 mL) to stop the reaction and stirred for another 2 h. Then, precipitation was filtered to give a yellow solid. Subsequently, the solid was dried overnight under vacuum to give compound **S1** (yield 78%). The ^{1}H NMR and ^{13}C NMR spectra of **S1** were shown below in Figures S16 and S17, respectively. ^{1}H NMR (500 MHz, $CDCl_3$) δ/ppm 9.60 (s, 1H), 7.29 (s, 2H), 3.29 (t, J = 5.0 Hz, 4H), 2.77 (t, J = 10.0 Hz, 4H), 1.97 (m, 4H). ^{13}C NMR (126 MHz, $CDCl_3$) δ/ppm 190.1, 147.9, 129.5, 124.1, 120.4, 50.0, 27.6, 21.3.

Preparation of compound S2

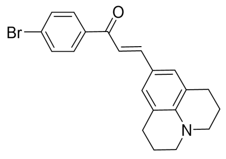

S2

Compound **S1** (2.00 g, 10 mmol), 4-bromoacetophenone (2.00 g, 10 mmol) and NaOH aqueous solution (20%, 10 mL) were slowly added into the 100 mL round bottom flask containing ethanol (20 mL). The mixture was stirred at room temperature for 24 h. Then, the reacted solution was poured into ice-water (100 mL) to quench the reaction and stirred for another 2 h. After that, precipitation was filtered to give a red solid. Subsequently, the red solid was dried overnight under vacuum to offer compound **S2** (yield 80%). The ^{1}H NMR and ^{13}C NMR spectra of **S2** were shown below in Figures S18 and S19, respectively. ^{1}H NMR (500 MHz, $CDCl_3$) δ/ppm 7.85 (d, J = 10.0 Hz, 2H), 7.69 (d, J = 20.0 Hz, 1H), 7.59 (d, J = 10.0 Hz, 2H), 7.18 (d, J = 20.0 Hz, 1H), 7.11 (s, 2H), 3.26 (t, J = 5.0 Hz, 4H), 2.76 (t, J = 5.0 Hz, 4H), 1.97 (m, 4H). ^{13}C NMR (126 MHz, $CDCl_3$) δ/ppm 189.3, 147.0, 145.5, 138.1, 131.6, 129.9, 128.4, 126.8, 121.3, 121.0, 114.9, 108.1, 105.9, 50.0, 27.7, 21.5. IT-TOF/MS: Calcd for $[M + H]^+$ 382.0728, found: 382.0804.

Preparation of compound S3

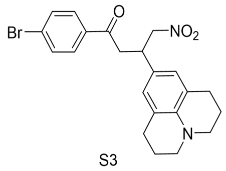

S3

Compound **S2** (1.00 g, 5 mmol), nitromethane (1.00 mL) and N, N-diisopropylethylamine (DIPEA, 1.0 mL, 5.5 mmol) were slowly added into the 100 mL round bottom flask containing methanol (20 mL). The mixture was stirred at 65 °C for 24 h. Then, the reacted solution was poured into saturated sodium chloride aqueous solution (10 mL) to stop the reaction, and ethyl acetate (30 mL) was added into solution for extraction. The combined organic fractions were dried over anhydrous Na_2SO_4, and filtered to afford the crude product which was purified *via* flash column chromatography (silica gel, EA/PE = 1/10) to afford the yellow solid (yield 67%). The ^{1}H NMR and ^{13}C NMR spectra of **S3** were shown below in Figures S20 and S21, respectively. ^{1}H NMR (500 MHz, $CDCl_3$) δ/ppm 7.76 (d, J = 15.0 Hz, 2H), 7.59 (d, J = 10.0 Hz, 2H), 6.63 (s, 2H), 4.72 (t, J = 5.0 Hz, 1H), 4.60 (t, J = 5.0 Hz,1H), 3.98 (t, J = 10.0 Hz, 1H). 3.38 (m, 2H). 3.10 (t, J = 5.0 Hz, 4H), 2.70 (t, J = 5.0 Hz, 4H), 1.97 (m, 4H). ^{13}C NMR (126 MHz, $CDCl_3$) δ/ppm 196.5, 142.5, 135.3, 132.0, 129.6, 128.6, 125.8, 125.4, 121.8, 80.0, 49.9, 41.9, 38.6, 27.7, 21.9, 14.8. IT-TOF/MS: Calcd for $[M + H]^+$ 443.0892, found: 443.0965.

Preparation of compound S4

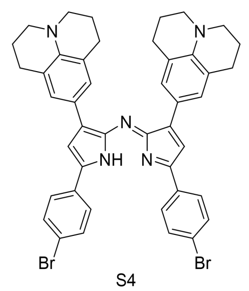

S4

Compound **S3** (1.00 g, 1.0 mmol) and ammonium acetate (1.16 g, 15.0 mmol) were added into a 100 mL round bottom flask containing n-butanol (20 mL), and stirred at 90 °C for 24 h. The reaction was cooled to room temperature, and the solvent was concentrated to 5 mL in vacuo and then filtered. The isolated solid washed with ethanol (2 × 5 mL) to afford the blue-black solid (yield 37%). The ^{1}H NMR and ^{13}C NMR spectra of **S4** were shown below in Figures S22 and S23, respectively. ^{1}H NMR (500 MHz, $CDCl_3$) δ/ppm 7.77 (d, J = 15.0 Hz, 4H), 7.63 (d, J = 10.0 Hz, 4H), 7.56 (s, 4H), 6.94 (s, 2H), 5.30 (s, 1H), 3.22 (t, J = 5.0 Hz, 8H), 2.76 (t, J = 10.0 Hz, 8H), 2.01 (m, 8H). ^{13}C NMR (126 MHz, $CDCl_3$) δ/ppm 156.1, 150.5, 143.9, 138.8, 131.8, 128.7, 125.5, 124.5, 123.3, 121.7, 115.8, 49.9, 27.9, 21.8, 1.0.

Preparation of compound S5

Under the protection of N_2 atmosphere, compound **S4** (80 mg, 0.11 mmol) and DIPEA (0.2 mL, 1.1 mmol) were added into a 100 mL round bottom flask containing dry DCM (20 mL). Then, $BF_3 \cdot Et_2O$ (0.28 mL, 2.0 mmol) was slowly added into the solution, and stirred at room temperature under dark for 24 h. Then, the solution was

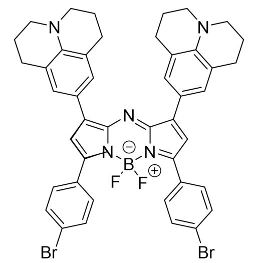

S5

diluted with ice-water (20 mL) and extracted with DCM (3 × 20 mL). The combined organic fractions were dried over anhydrous Na_2SO_4, filtered and concentrated to afford the crude residue. The crude product was purified *via* flash column chromatography (silica gel, DCM/PE = 1/1) to afford the metallic shiny solid (yield 84%). The ^{1}H NMR and ^{13}C NMR spectra of **S5** were shown below in Figures S24 and S25, respectively. ^{1}H NMR (500 MHz, $CDCl_3$) δ/ppm 7.86 (d, J = 10.0 Hz, 4H), 7.60 (s, 4H), 7.56 (d, J = 10.0 Hz, 4H), 6.69 (s, 2H), 3.30 (t, J = 5.0 Hz, 8H), 2.77 (t, J = 5.0 Hz, 8H), 2.00 (m, 8H). ^{13}C NMR (126 MHz, $CDCl_3$) δ/ppm 156.1, 150.5, 143.9, 138.8, 131.8, 128.7, 125.5, 124.5, 123.3, 121.7, 115.8, 49.9, 30.9, 29.7, 27.9, 24.4, 21.8, 11.2, 1.4. MALDI-TOF/MS: $[M]^+$ calcd: 845.1530, found: 845.1960.

Preparation of compound SW1

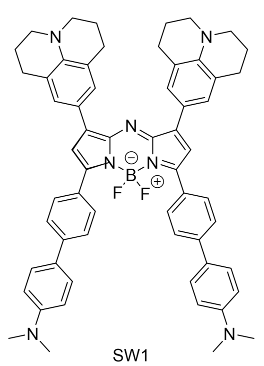

SW1

Under the protection of N_2 atmosphere, compound **S5** (50 mg, 0.06 mmol), 4-(dimethylamino) phenylboronic acid (54 mg, 0.12 mmol), $Pd(PPh_3)_4$ (115 mg, 0.1 mmol), K_2CO_3 (1.03 g, 7.5 mmol) and dioxane/H_2O (10 mL, 5 : 1) were added into a 50 mL round bottom flask. The mixture was stirred at 90 °C for 24 h. Then, the reaction was cooled to room temperature and the solvent was concentrated to 5 mL, and filtered to afford the crude residue. The crude product was purified *via* flash column chromatography (silica gel, MeOH/DCM = 1/50) to afford the blue-black solid (yield 61%). The ^{1}H NMR and ^{13}C NMR spectra of **SW1** were shown below in Figures S26 and S27, respectively. ^{1}H NMR (500 MHz, $CDCl_3$) δ/ppm 8.11 (d, J = 10.0 Hz, 4H), 7.65 (d, J = 10.0 Hz, 8H), 7.59 (d, J = 10.0 Hz, 4H), 6.83 (d, J = 10.0 Hz, 4H), 6,79 (s, 2H), 3.28 (t, J = 10.0 Hz, 8H), 3.01 (s, 12H), 2.79 (t, J = 5.0 Hz, 8H), 2.02 (m, 8H). ^{13}C NMR (126 MHz, $CDCl_3$) δ/ppm 156.2, 150.2, 145.6, 143.9, 143.2, 142.4, 129.8, 128.6, 127.8, 125.9, 121.2, 114.8, 112.7, 67.1, 50.2, 40.5, 29.7, 28.0, 21.8. MALDI-TOF/MS: $[M]^+$ calcd: 925.4815, found: 925.8859.

Preparation of compound SW2

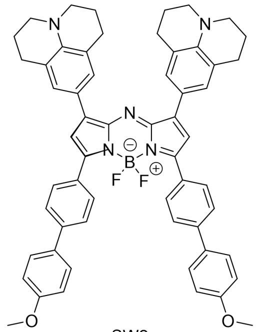

SW2

Blue-green solid was prepared according to a similar procedure to **SW1** by using p-benzyloxy-4-boronic acid (yield 60%). The ^{1}H NMR and ^{13}C NMR spectra of **SW2** were shown below in Figures S28 and S29, respectively. ^{1}H NMR (500 MHz, $CDCl_3$) δ/ppm 8.11 (d, J = 10.0 Hz, 4H), 7.63 (d, J = 5.0 Hz, 8H), 7.60 (t, J = 10.0 Hz, 4H), 6.99 (d, J = 5.0 Hz, 4H), 6.81 (s, 2H), 3.86 (s, 6H), 3.29 (t, J = 5.0 Hz, 8H), 2.79 (t, J = 5.0 Hz, 8H), 2.00 (m, 8H). ^{13}C NMR (126 MHz, $CDCl_3$) δ/ppm 159.4, 156.1, 145.6, 144.0, 143.5, 141.9, 133.1, 131.0, 129.8, 128.7, 128.2, 126.5, 121.2, 120.8, 114.3, 55.4, 50.2, 28.0, 21.8, 14.1. MALDI-TOF/MS: $[M]^+$ calcd: 899.4182, found: 899.7998.

Preparation of compound SW3

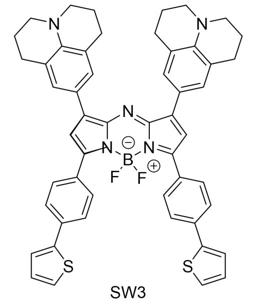

SW3

Blue-green solid was prepared according to a similar procedure to **SW1** by using thiophene-4-boronic acid (yield 65%). The ^{1}H NMR and ^{13}C NMR spectra of **SW3** were shown below in Figures S30 and S31, respectively. ^{1}H NMR (500 MHz, $CDCl_3$) δ/ppm 8.07 (d, J = 5.0 Hz, 4H), 7.69 (d, J = 5.0 Hz, 4H), 7.63 (s, 4H), 7.39 (s, 2H), 7.31 (d, J = 5.0 Hz, 2H), 7.10 (d, J = 5.0 Hz, 2H), 6.80 (s, 2H), 3.29 (t, J = 5.0 Hz, 8H), 2.79 (t, J = 5.0 Hz, 8H), 2.01 (m, 8H). ^{13}C NMR (126 MHz, $CDCl_3$) δ/ppm 156.2, 150.2, 145.6, 143.9,143.2, 142.4, 130.2, 129.8, 128.6, 127.8, 125.9, 121.2, 114.8, 112.7, 67.1, 50.2, 40.5, 29.7, 28.0, 21.8. MALDI-TOF/MS: $[M]^+$ calcd: 851.3099, found: 851.7133.

Preparation of compound SW4

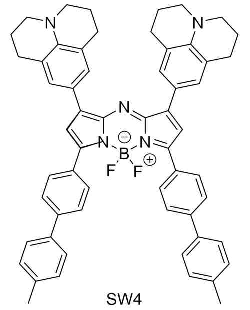
SW4

Blue-black solid was prepared according to a similar procedure to **SW1** by using p-benzyl-4-boronic acid (yield 24%). The ^{1}H NMR and ^{13}C NMR spectra of **SW4** were shown below in Figures S32 and S33, respectively. ^{1}H NMR (500 MHz,$CDCl_3$) δ/ppm 8.66 (d, J = 5.0 Hz, 2H), 8.38 (d, J = 5.0 Hz, 2H), 7.95 (d, J = 10.0 Hz, 2H), 7.70 (d, J = 10.0 Hz, 2H), 7.64 (d, J = 10.0 Hz, 2H), 7.51 (s, 4H), 7.42 (s, 2H), 7.33 (d, J = 10.0 Hz, 2H), 7.05 (d, J = 5.0 Hz, 2H), 6.96 (s, 1H), 6.55 (s, 1H), 3.22 (t, J = 5.0 Hz, 8H), 2.71 (t, J = 10.0 Hz, 8H), 2.57 (s, 6H), 1.98 (m, 8H). ^{13}C NMR (126 MHz, $CDCl_3$) δ/ppm 156.1, 145.7, 143.5, 142.3, 137.7, 137.5, 131.5, 130.7, 129.8, 129.5, 128.8, 128.7, 127.0, 126.8, 121.3, 121.2, 114.7, 114.1, 49.4, 29.7, 28.1, 21.8, 21.2. MALDI-TOF/MS: $[M]^+$ calcd: 867.4284, found: 867.3991.

Preparation of compound SW5

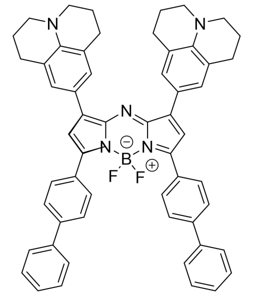
SW5

Blue-black solid was prepared according to a similar procedure to **SW1** by using phenyl-4-boronic acid (yield 36%). The ^{1}H NMR and ^{13}C NMR spectra of **SW5** were shown below in Figures S34 and S35, respectively. ^{1}H NMR (500 MHz, $CDCl_3$) δ/ppm 8.14 (d, J = 10.0 Hz, 4H), 7.67 (t, J = 10.0 Hz, 12H), 7.45 (t, J = 15.0 Hz, 4H), 7.36 (t, J = 10.0 Hz, 2H), 6.82(s, 2H), 3.29 (t, J = 5.0 Hz, 8H), 2.79 (t, J = 5.0 Hz, 8H), 2.00 (m, 8H). ^{13}C NMR (126 MHz, $CDCl_3$) δ/ppm 156.1, 145.7, 144.1, 143.6, 142.3, 140.6, 131.7, 129.8, 128.8, 127.6, 127.2, 127.0, 121.3, 120.8, 114.6, 50.2, 29.7, 28.1, 21.8. MALDI-TOF/MS: $[M]^+$ calcd: 839.3971, found: 839.5818.

Preparation of compound SW6

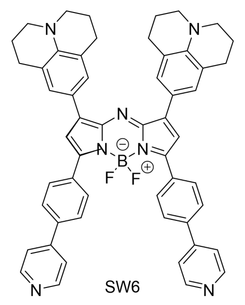
SW6

Blue-black solid was prepared according to a similar procedure to **SW1** by using pyridine-4-boronic acid (yield 61%). The ^{1}H NMR and ^{13}C NMR spectra of **SW6** were shown below in Figures S36 and S37, respectively. ^{1}H NMR (500 MHz, $CDCl_3$) δ/ppm 8.67 (d, J = 5.0 Hz, 4H), 8.13 (d, J = 5.0 Hz, 4H), 7.70 (s, 4H), 7.64 (d, J = 5.0 Hz, 4H), 7.55 (d, J = 5.0 Hz, 4H), 6.78 (s, 2H) 3.31 (t, J = 5.0 Hz, 8H), 2.78 (t, J = 5.0 Hz, 8H), 2.01 (m, 8H). ^{13}C NMR (126 MHz, $CDCl_3$) δ/ppm 153.1, 150.2, 148.4, 148.2, 147.6, 143.5, 141.0, 138.1, 135.9, 135.0, 134.9, 131.6, 130.3, 128.0, 127.5, 126.9, 121.8, 121.5, 121.3, 121.1, 111.4, 51.4, 31.9, 30.2, 29.7, 27.9, 22.7, 22.1, 13.4. MALDI-TOF/MS: $[M]^+$ calcd: 841.3876, found: 841.7387.

Preparation of compound SW7

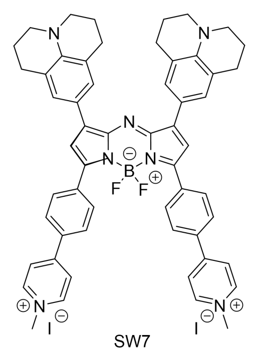
SW7

Under the protection of N_2 atmosphere, **SW6** (50 mg, 0.06 mmol) and methyl iodide (17 mg, 0.12 mmol) were added into a 100 mL round bottom flask containing dry DCM (25 mL). The mixture was stirred at room temperature for 36 h. Then, the reacted solution was diluted with ice-water to stop the reaction and continue to stir for another 2 h. A blue-green solid be obtained by filtration without any further purification (yield 63%). The ^{1}H NMR and ^{13}C NMR spectra of **SW7** were shown below in Figures S38 and S39, respectively. ^{1}H NMR (500 MHz, d_6-DMSO) δ/ppm 9.06 (d, J = 10.0 Hz, 4H), 8.63 (d, J = 10.0 Hz, 4H), 8.33 (m, 8H), 7.58 (s, 4H), 7.55 (s, 2H), 4.36 (s, 6H), 3.24 (t, J = 10.0 Hz, 8H), 2.69 (d, J = 10.0 Hz, 8H), 1.92 (s, 8H). ^{13}C NMR (126 MHz, d_6-DMSO) δ/ppm 153.4, 151.4, 146.6, 143.7, 135.2, 129.4, 128.1, 123.0, 119.8, 63.3, 55.4, 53.4, 49.8, 47.1, 40.0, 27.1, 23.1, 20.2, 15.4. MALDI-TOF/MS: $[M - 2I]^{2+}$ calcd: 871.433, found: 871.223.

Preparation of compound SW8

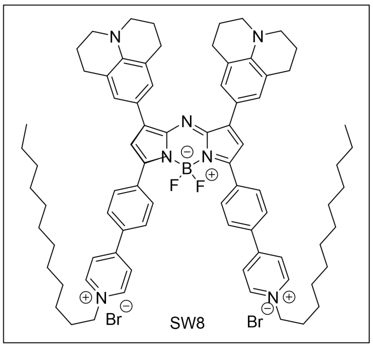

Under the protection of N_2 atmosphere, **SW6** (50 mg, 0.06 mmol) and 1-bromododecane (30 mg, 0.12 mmol) were added into sealed tube containing $CHCl_3$ (10 mL), and stirred at 100 °C for 24 h. The reaction was cooled to room temperature and the solvent was concentrated in vacuo. Finally, the resulting mixture was purified *via* flash column chromatography (silica gel, MeOH/DCM = 1/25) to afford the blue-black solid (yield 16%). The ^{1}H NMR and ^{13}C NMR spectra of **SW8** were shown below in Figures S40 and S41, respectively. ^{1}H NMR (500 MHz, $CDCl_3$) δ/ppm 9.08 (s, 4H), 8.09 (t, J = 5.0 Hz, 8H), 7.67 (s, 4H), 7.57 (s, 4H), 6.96 (s, 2H), 4.61 (s, 4H), 3.32 (s, 8H), 2.77 (s, 8H), 2.00 (s, 8H), 1.84 (s, 4H), 1.21 (t, J = 20.0 Hz, 36H), 0.86 (t, J = 5.0 Hz, 6H). ^{13}C NMR (126 MHz, $CDCl_3$) δ/ppm 154.4, 153.6, 151.8, 146.8, 144.8, 144.5, 143.4, 136.1, 133.6, 130.4, 129.2, 128.0, 124.8, 124.7, 121.7, 120.5, 115.0, 61.1, 50.3, 31.9, 31.6, 29.6, 29.5, 29.3, 29.1, 28.0, 27.2, 26.2, 25.3, 22.7, 21.6, 14.1. MALDI-TOF/MS: $[M - 2Br]^{2+}$ calcd: 1180.481, found: 1180.482.

2. Photophysical properties of SW1-8

NIR absorption and fluorescence emission spectrum: The target aza-BODIPY based NIR-II PTAs (**SW1-8**) were dissolved in different solvents (1×10^{-5} mol L^{-1}) for the experiment. The test solution was added into the cuvette, and then use the Hitachi UH5700-spectrophotometer to record the NIR absorption spectra of **SW1-8** in different solvents at room temperature (Fig. S1). Then, the fluorescence emission curve (λ_{ex} = 808 nm) of **SW1-8** were recorded in the same solvents (Fig. S2). Normalized absorption and fluorescence emission spectra of **SW1-8** in DMSO were shown in Fig. 2c in the maintext.

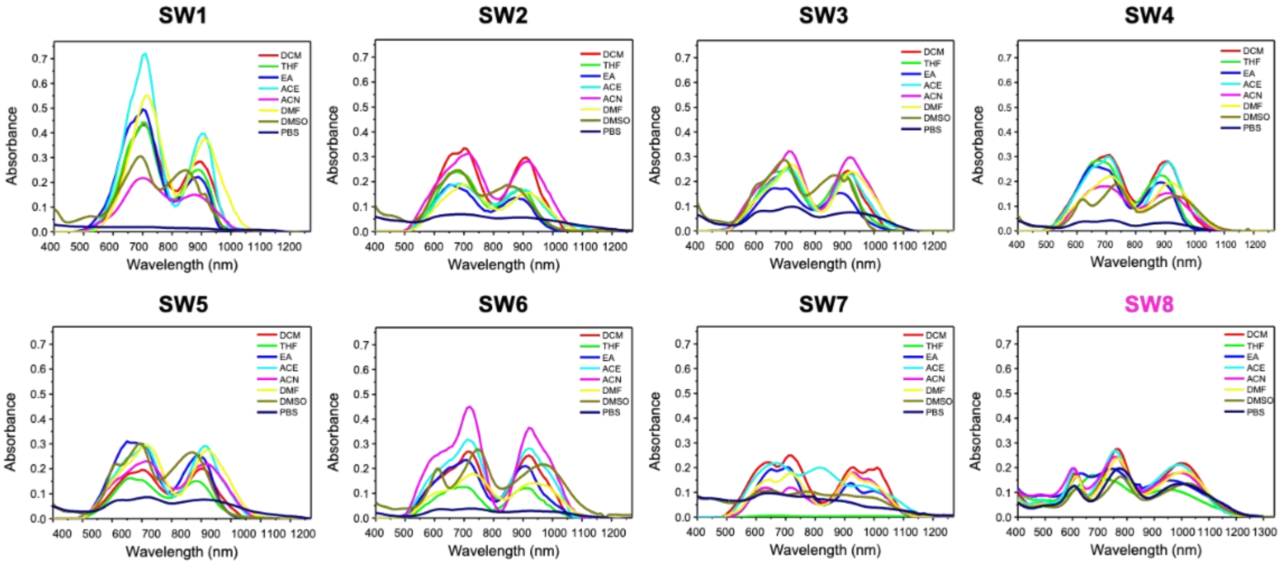

Fig. S1. The absorption spectra of **SW1-8** in different organic solvents (1×10^{-5} mol L^{-1}).

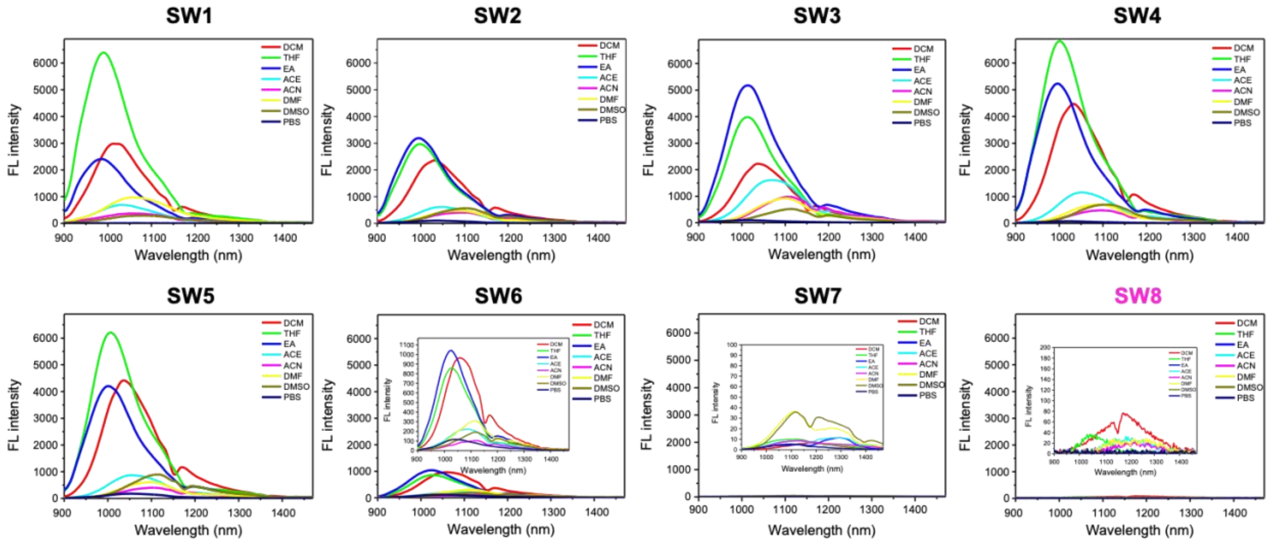

Fig. S2. The emission spectra (λ_{ex} = 808 nm) of **SW1-8** in different organic solvents (1×10^{-5} mol L^{-1}). Inserts are the enlarged spectra from 900 to 1475 nm for corresponding compounds.

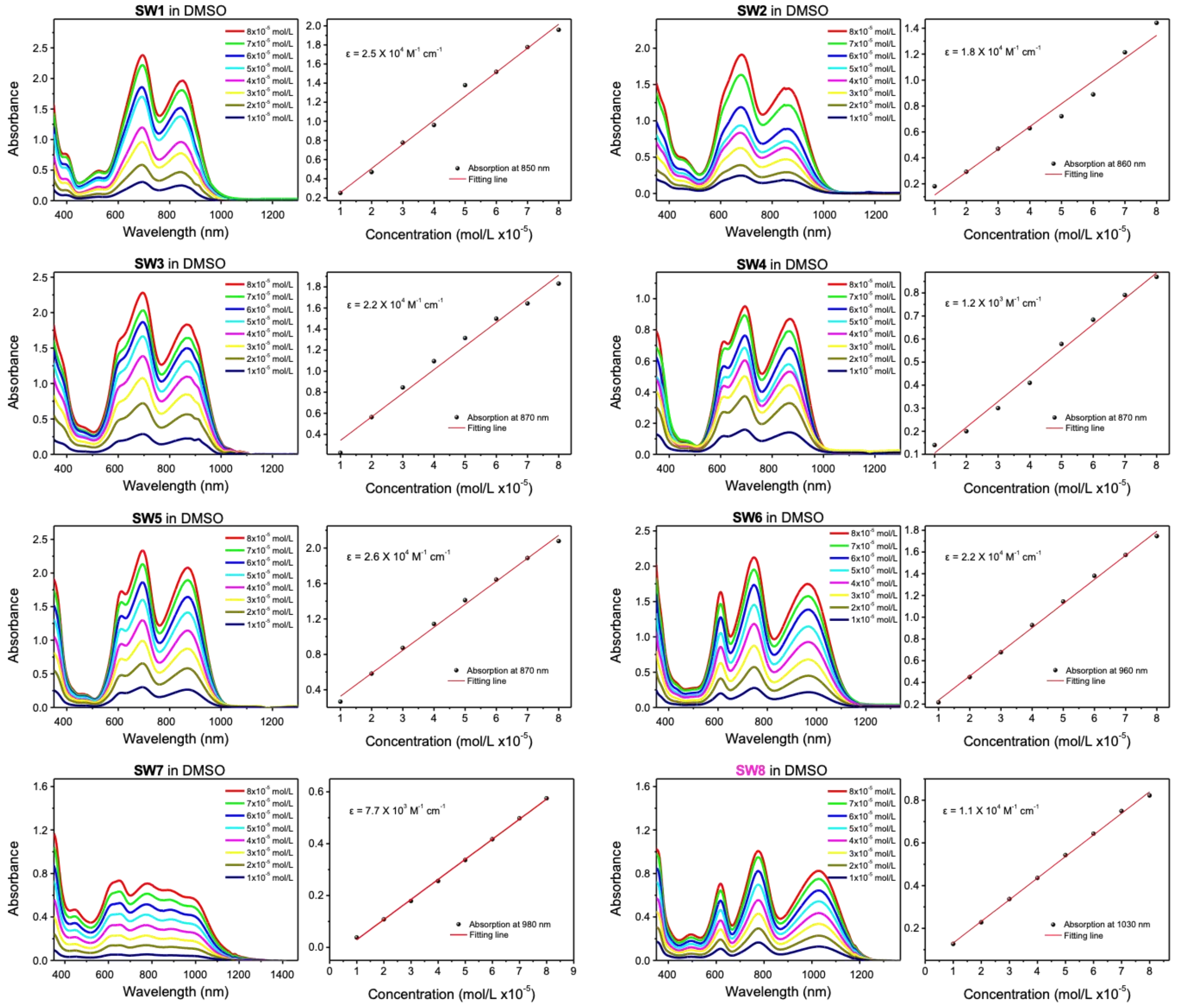

Fig. S3. Concentration dependence of the absorption of **SW1-8** in DMSO. The plot of optical density at the

absorption maxima versus concentration. The straight line is a linear least-squares fit to give the molar extinction coefficient of **SW1-8** at the absorption maxima.

The quantum yield of the **SW1-8** was determined according the formula:

$$\Phi_X = \Phi_{st}(\frac{S_X}{S_{st}})(\frac{A_{st}}{A_X})(\frac{n^2{}_X}{n^2{}_{st}})$$

to where Φ_{st} is the quantum yield of the standard, *S* is the area under the emission spectra, *A* is the absorbance at the excitation wavelength, and *n* is the refractive index of the solvent used. *X* subscript denotes sample, and *st* means standard. IR1061 (Φ_f = 0.017 in DCM) was chosen as the standard (*1*).

Table S1. Photophysical properties of **SW1-8** in different solvents.

Compounds	Solvent	λ_{abs}/nm	λ_{em}/nm	ε[a]	Φ%[b]	Brightness
**SW1**	DCM	900	1020	28000	8.33	2332
	THF	890	990	25000	19.31	4828
	EA	890	980	22000	7.99	1758
	ACE	910	1030	40000	3.36	1344
	ACN	880	1070	15000	1.81	272
	DMF	910	1060	38000	3.87	1471
	DMSO	850	1080	25000	1.12	280
	PBS	/	/	/	/	/
**SW2**	DCM	910	1030	30000	9.74	2922
	THF	890	1000	17000	11.97	2035
	EA	880	1000	14000	14.28	1999
	ACE	890	1050	17000	2.50	425
	ACN	910	1090	28000	1.55	434
	DMF	920	1100	16000	2.79	446
	DMSO	860	1100	18000	1.94	349
	PBS	920	/	5600	/	/
**SW3**	DCM	910	1040	24000	12.33	2959
	THF	900	1010	23000	16.05	3692
	EA	890	1010	15000	23.30	3495
	ACE	920	1070	23000	9.08	2088
	ACN	920	1110	30000	5.11	1533
	DMF	920	1100	24000	5.41	1298
	DMSO	870	1110	22000	1.64	361
	PBS	940	/	15300	/	/
**SW4**	DCM	900	1030	28000	16.88	4726
	THF	890	1000	22000	22.27	4899
	EA	890	1000	20000	19.50	3900
	ACE	910	1050	28000	5.97	1672
	ACN	910	1100	15000	2.62	393
	DMF	920	1090	20000	4.17	834
	DMSO	870	1100	14000	4.93	690
	PBS	910	/	3300	/	/
**SW5**	DCM	940	1040	20000	19.93	3986
	THF	880	1010	15000	27.32	4098
	EA	890	1000	26000	13.72	3567
	ACE	910	1060	30000	4.31	1293
	ACN	920	1100	22000	1.60	352
	DMF	920	1100	28000	2.97	832
	DMSO	870	1120	26000	2.32	603
	PBS	920	1060	7600	1.28	97

**SW6**	DCM	920	1060	25000	6.27	1568
	THF	910	1030	12000	9.21	1105
	EA	910	1030	21000	6.57	1380
	ACE	920	1090	28000	1.34	375
	ACN	920	1120	36000	1.40	504
	DMF	950	1120	14000	1.84	258
	DMSO	960	1120	22000	0.91	200
	PBS	980	1140	2700	2.30	62
**SW7**	DCM	1010	1180	20000	0.01	2
	THF	/	/	/	/	/
	EA	1000	1300	14000	0.02	3
	ACE	980	1300	13000	0.03	4
	ACN	980	1130	16000	0.03	5
	DMF	980	1110	14000	0.09	13
	DMSO	990	1110	7700	0.08	6
	PBS	1000	1100	3900	0.02	1
**SW8**	DCM	1000	1180	22000	0.10	22
	THF	950	1050	11000	0.09	10
	EA	950	/	15000	/	/
	ACE	990	1200	22000	0.05	11
	ACN	1000	1230	18000	0.03	5
	DMF	1010	1230	18000	0.05	9
	DMSO	1030	1230	12000	0.02	2
	PBS	1000	/	13000	/	/

[a] Extinction coefficient; [b] Fluorescence quantum yield; Brightness = ε × Φ.

3. Preparation, characterization and photophysical properties of SW1-8@NPs

To prepare NIR-II aza-BODIPY photothermal agents NPs, **SW1-8** (1 mg) and DSPE-PEG_{5000} (5 mg) were dissolved in THF (1 mL) and mixed uniformity under ultrasound to work as the stock solution. Then above stock solution was added dropwise into deionized water (9 mL) and dispersed under ultrasonic cell disruptor. After the organic solvents evaporated in the fume hood, the resultant NPs were harvested by ultrafiltration filter (Millipore) and purified with 0.45 μm filtration. The resulted NPs were stored in 4 °C freezer for the following usage.

There are the following steps to calibrate the concentration of nanoparticles and calculate the loading capacity:

1) The molar absorption coefficient of SW1-8 in DMSO was determined and denoted as $\varepsilon_{SW1\text{-}8}$.

2) 1 mg SW1-8 was weighed and dissolved in THF, and then coated into nanoparticles with DSPE-PEG5000. Nanoparticles solution of unknown concentration was obtained after coating, and its constant volume was 10 mL.

3) we take 2 mL solution and test its absorbance in water, denoted as A_{NPs}. The remaining solution was divided into 3 parts of 2 mL each and freeze-drying, then DMSO (2 mL) was added to dissolve the solution. The absorbance in DMSO was tested, and the average value was denoted as A.

4) $A = \varepsilon_{DMSO,\ NPs} \times C_{NPs}$, $\varepsilon_{DMSO,\ NPs} \approx \varepsilon_{SW1\text{-}8}$, C_{NPs} are obtained after calculation. Loading capacity = $C_{NPs} \times 10 \times 100\%$.

5) SW1-8@NPs solution can be configured with 1mg/mL depending on the loading capacity.

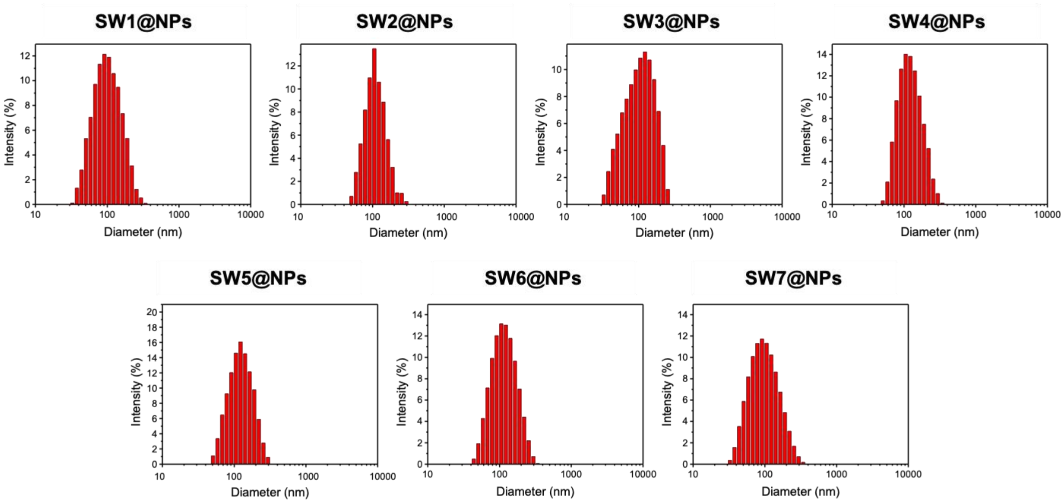

Fig. S4. DLS measurements of **SW1-7@NPs**. The data of **SW8@NPs** was shown in Fig. 3A in the maintext.

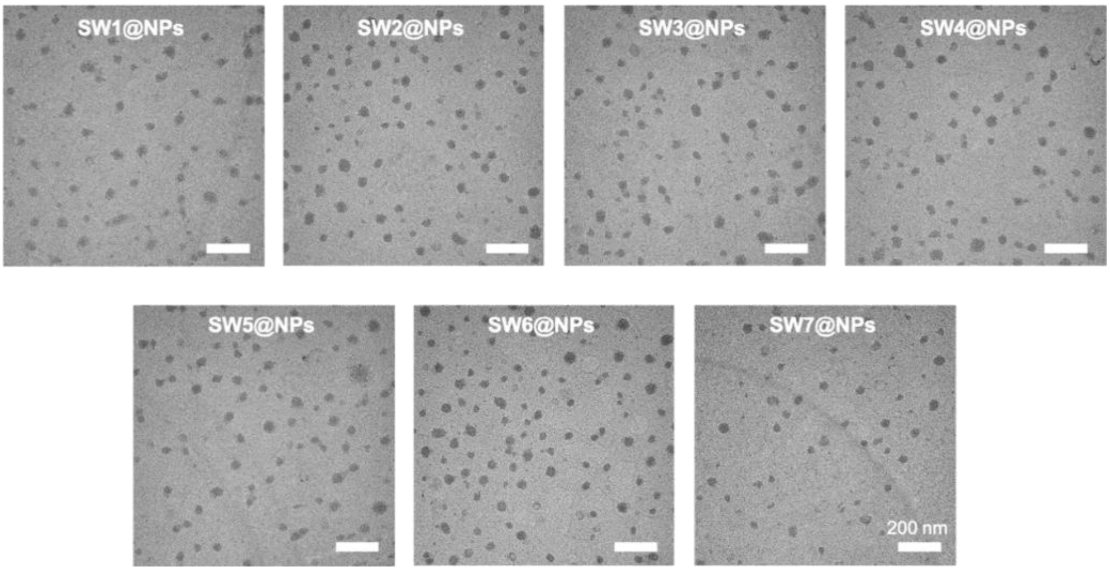

Fig. S5. TEM measurements of **SW1-7@NPs**. The data of **SW8@NPs** was shown in Fig. 3a in the maintext. Scale bar = 200 nm.

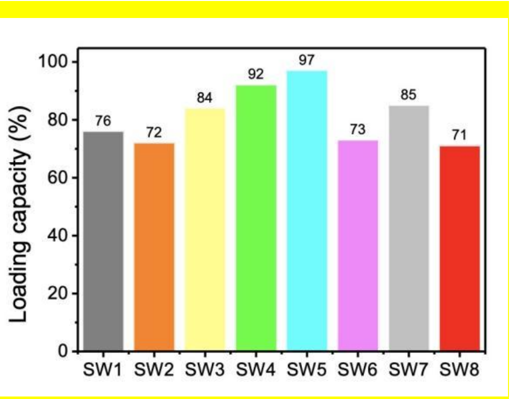

Fig. S6. Loading capacity of **SW1-8**.

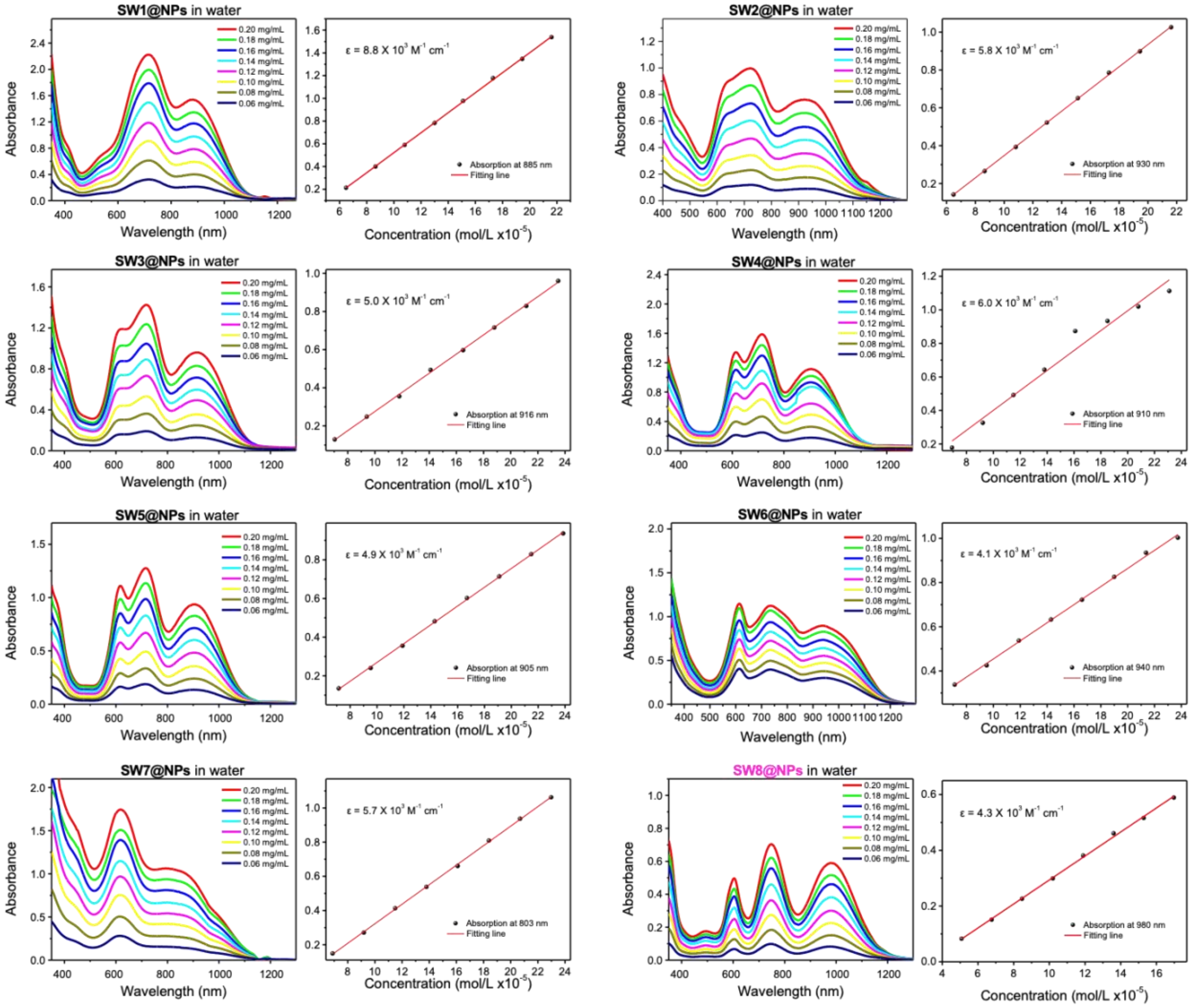

Fig. S7. Concentration dependence of the absorption of **SW1-8@NPs** in water. The plot of optical density at the absorption maxima versus concentration. The straight line is a linear least-squares fit to give the molar extinction coefficient of **SW1-8@NPs** at the absorption maxima.

4. Ultrafast spectroscopic and kinetic measurements

To prepare NIR-II aza-BODIPY photothermal agents NPs, **SW1-8** (1 mg) and DSPE-PEG$_{5000}$ (5 mg) were dissolved in THF (1 mL) and mixed uniformity under ultrasound to work as the stock solution. Then above stock solution was added dropwise into deionized water (9 mL) and dispersed under ultrasonic cell disruptor. After the organic solvents evaporated in the fume hood, the resultant NPs were harvested by ultrafiltration filter (Millipore) and purified with 0.45 μm filtration. The resulted NPs were stored in 4 °C freezer for the following usage.

Table S2. Fitting parameters for the representative wavelengths within the ground-state bleaching (GSB) region.

Compounds	τ_1(ps)	A_1	τ_2 (ps)	A_2	τ_3 (ps)	A_3
**SW5@NPs**	/	/	5	73.40%	56	26.60%
**SW6@NPs**	/	/	2.5	72.80%	43	27.20%
**SW8@NPs**	0.077	81.50%	3.11	8.30%	13.3	10.20%

The decays were fitted with the following function: $I = A_1e^{-t/\tau1} + A_2e^{-t/\tau2} + A_3e^{-t/\tau3}$. A_1, A_2, and A_3 denote the fraction of excited population of the associated component.

5. Photothermal properties measurement

To examine the photothermal performance of the prepared NPs, **SW1-8@NPs** aqueous solution with various concentrations and pure water as control were placed into the 808/1064 nm laser (Changchun New Industries Optoelectronics Tech. Co., Ltd., Changchun, China) irradiation from for 10 min, respectively. The temperature profiles and images were precisely recorded with a thermal imaging camera (FLIR E40). In addition, different laser power intensity was employed to investigate the photothermal performance of NPs.

To evaluate the photothermal conversion efficiency (PCE), the temperature variations of NPs aqueous solution (200 μg mL^{-1}) were recorded upon 808/1064 nm laser irradiation (0.23 W cm^{-2}) for 10 min to achieve the maximum plateau of temperature, and the 808/1064 nm laser was turned off for natural cooling to room temperature. Thus, PCE can be calculated by referring to the following Equation (1):

$$PCE = \frac{hS(T_{Max} - T_{Surr}) - Q_{Dis}}{I(1 - 10^{-A})} \text{...... (1);}$$

Where h indexes the heat transfer coefficient; S represents the surface area of the used holder; TMax and TSurr denote the maximum steady-state temperature and room temperature of the ambient environment, respectively; QDis is the heat wastage form the light loss of the solvent and holder, and the QDis was determined using pure water; I indexes laser intensity (0.23 W cm-2), and A represents the 808/1064 nm absorbance of NPs. hS can be calculated referring to the following Equation (2):

$$\tau_s = \frac{m_D c_D}{hS} \text{...... (2);}$$

Where mD and cD index the solution mass and heat capacity (4.2 J g-1) of pure water used as the solvent, respectively. As noted, τ_s can be calculated referring to the following Equation (3):

$$t = -\tau_s \ln\left(\frac{T_{RT} - T_{Surr}}{T_{Max} - T_{Surr}}\right) \text{......(3);}$$

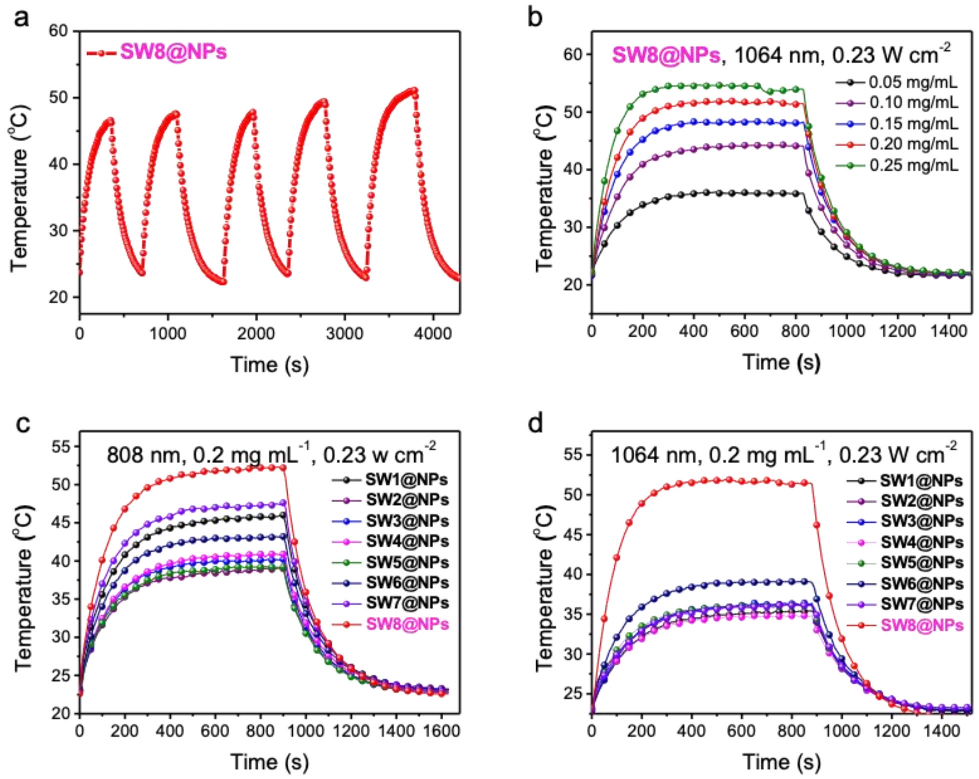

Fig. S8. (a) Photothermic heating curves of the **SW8@NPs** in water with different concentrations under 1064 nm laser irradiation (0.23 W cm^{-2}). (b) Photothermic stability of **SW8@NPs** upon 1064 nm laser irradiation (0.2 mg mL^{-1}, 0.23 W cm^{-2}) for five on/off cycles. Photothermic heating curves of the **SW8@NPs** in water under (c) 808 nm laser irradiation or (d)1064 nm laser 0.23 W cm^{-2} for 15 min followed by cooling to room temperature.

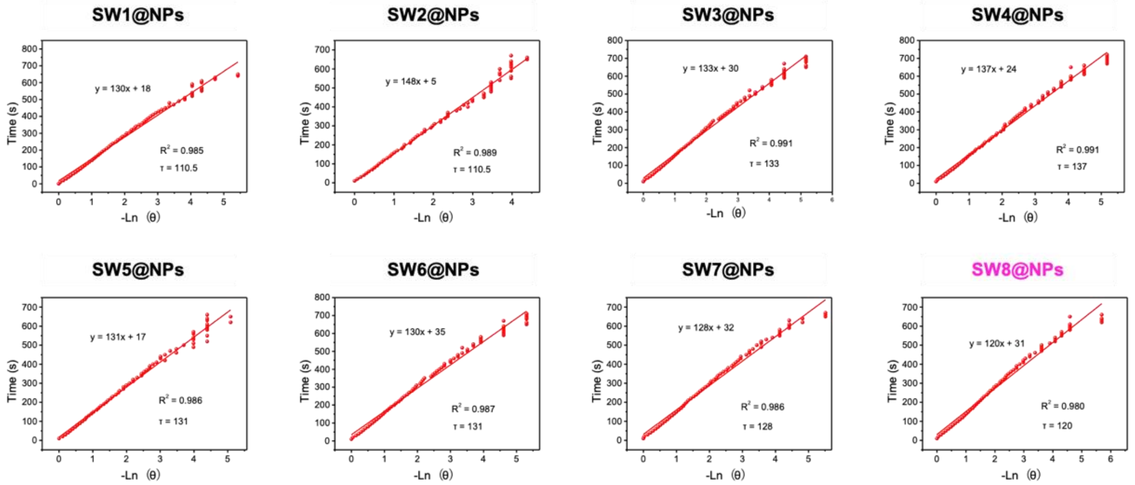

Fig. S9. Linear correlation of the cooling times versus negative natural logarithm of driving force temperatures (808 nm laser irradiation).

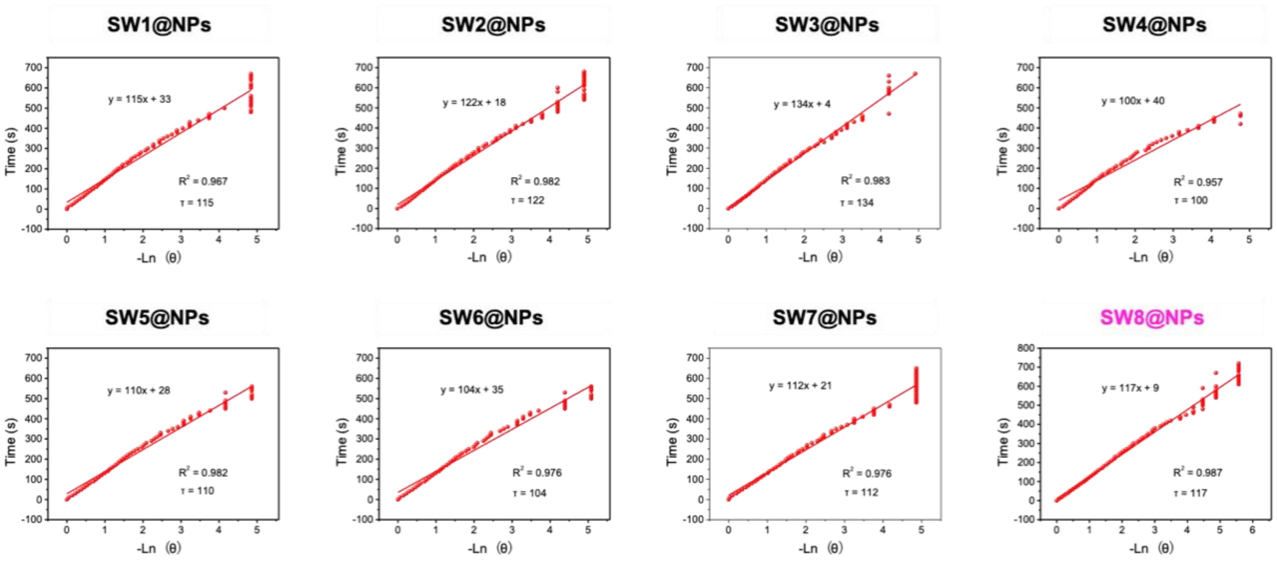

Fig. S10. Linear correlation of the cooling times versus negative natural logarithm of driving force temperatures (1064 nm laser irradiation).

Table S3. Summary of NIR-II absorbing small organic photothermal agents (SOPTAs).

SOPTAs	λ_{abs}/nm	λ_{em}/nm	ε_{1064}[a]	Φ%[b]	PCE%	Power[c] (W cm^{-2})	application	Ref.
IR-SS NPs	1120	NA	NA	NA	77	1	PA-guided PTT[d]	(2)
BAF4 NPs	1003	NA	~7000	NA	80	0.75	PA-guided PTT[d]	(3)
CSMN2	878	NA	7170	NA	31.60	1	PA-guided PTT[d]	(4)
Polipo-IR NPs	~970	NA	NA	NA	45.25	1.33	PA-guided PTT[d]	(5)
Nano-BFF	700	NA	222000	NA	34.30	1.5	PA-guided PTT[e]	(6)

[a] Extinction coefficient; [b] Fluorescence quantum yield; [c] 1064 nm laser; [d] Subcutaneous tumor PTT; [e] Deep tumor PTT.

6. *In vitro* experiments

Cell lines and cell culture

143B (human osteosarcoma cells), HUVEC (human umbilical vein endothelial cells), HepG2 (human epatic carcinoma cells), L02 (Human normal hepatocytes), Hela (human cervix carcinoma cells), U87 (Human astroglioma cells), and hCMEC (Human cardiac microvascular endothelial cells) were obtained from American Type Culture Collection. Cells were cultured under standard conditions (95% humidity, 5% CO_2, 37 °C) and harvested by 0.5% (w/v) trypsin-EDTA solution. Before undergoing further experiments, cells were seeded in 96- or 6-well plates at a density of 2×10^4 cells/cm^2 in DMEM medium (Gibco) supplemented with 10% fetal bovine serum (Gibco), 100 U/ml penicillin, and 100 µg/ml streptomycin (HyClone).

Cell viability assay

143B, HUVEC, HepG2, L02, Hela, hCMEC, and U87 cells in 96-well plates were treated with **SW8@NPs** at different concentrations (0, 2, 5, 10, 20, 50, 100 µg mL^{-1}) in serum-free medium. After incubation for 4 h, treated cells were illuminated with 808 nm or 1064 nm laser at a power density of 0.23 W cm^{-2} for 5 min. Cell viability was

determined using PrestoBlue Cell Viability Reagent (Invitrogen) after incubation for an additional 4 h. Briefly, PrestoBlue reagent and DMEM were prepared in a ratio of 1 : 9 and 100 μL was added to each well. Plates were incubated at 37°C with 5% CO_2 for 1 h and the absorbance was recorded at 570 nm using a microplate reader (TECAN SPARK). The results were shown in Fig. 4a in maintext and Fig. S10 in supporting information. Cell viability fractions were calculated by correcting the extinction of treated cells by the extinction of control cells.

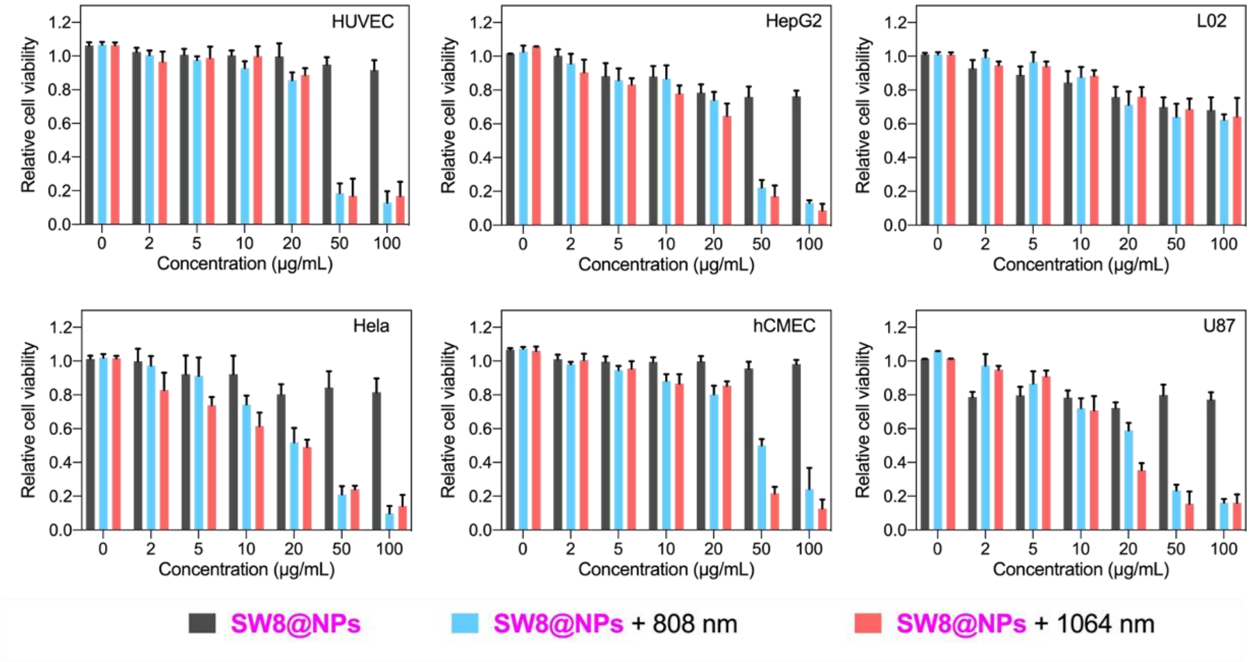

Fig. S11. Cell viabilities of **SW8@NPs** in 143B, HUVEC, HepG2, L02, Hela, hCMEC, and U87 cells at diverse concentrations without or with 808 nm/1064 nm laser (0.23 W cm^{-2} for 808 nm and 1064 nm), respectively. The data of 143B was shown in Fig. 4a in the maintext.

Table S4. Antibody information.

Antibodies	Company	Cat No	Host	Cross-reaction
Gasdermin D	Cell Signaling Technology	CST 39754	Rabbit	Mouse/Rat/Human
PARP1	Cell Signaling Technology	CST 9542S	Rabbit	Mouse/Rat/Human
Caspase-9	Cell Signaling Technology	CST 9508	Mouse	Mouse/Rat/Human
Caspase-3	Cell Signaling Technology	CST 14220	Rabbit	Mouse/Rat/Human
Caspase-1	Cell Signaling Technology	CST 24232	Rabbit	Mouse
Cleaved cas-1	Cell Signaling Technology	CST 89332	Rabbit	Mouse
Bax	Abcam	Ab32503	Rabbit	Mouse/Rat/Human
Bcl2	Abcam	Ab182858	Rabbit	Mouse/Human
Tubulin	Abcam	Ab7291	Mouse	Mouse/Human

Statistical analysis

All the experimental data were analyzed with GraphPad Prism (GraphPad Software, San Diego, CA). Normally distributed data sets were compared using a one-way ANOVA followed by Dunnet's post-hoc test where appropriate. A P-value of ≤ 0.05 was considered statistically significant. Data were expressed as mean ± SD.

7. Stability of SW8@NPs

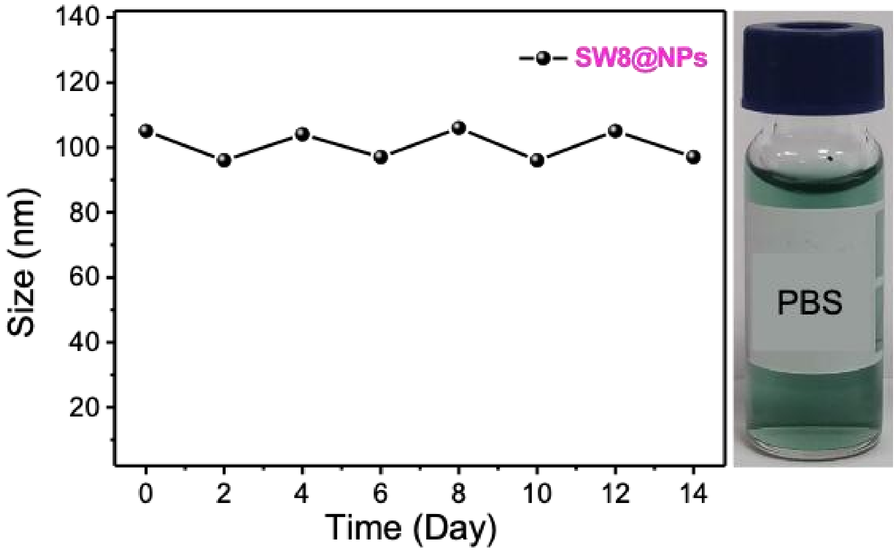

Fig. S12. Cell The stability of **SW8@NPs** storage at 4 °C for 14 days.

8. *In vivo* experiments

Animal model of osteosarcoma

Female BALB/c nude mice (4-6 weeks, 16.0-17.0 g) were purchased from Beijing Weitong Lihua Experimental Animal Technology Co., Ltd. (Beijing, PR China). All the animal studies were performed following the Guidelines for the Care and Use of Laboratory Animals of the Chinese Animal Welfare Committee and the protocol approved by the Animal Health and Use Committee of Northwestern Polytechnical University (202201035). 143B cells (3.0×10^7/mL) were used to develop the osteosarcoma mice model. A 1.0 mL syringe was applied to penetrate the tibial plateau into the medullary cavity and 30.0 μL of cells in PBS were injected into the bone marrow cavity of the left tibia. Tumors were allowed to grow for approximately 18 days to reach a volume of about 80-100 mm^3.

Fluorescence imaging assessments

The *in vivo* fluorescence imaging signals of **SW8@NPs** (100 μL, 1 mg mL^{-1} by tail vein injection) were collected with the excitation wavelengths at 808 nm. The fluorescence images and signal intensities at tumor sites of the mice were monitored at various time intervals post-injection (0, 0.5, 1, 2, 6, 12, 24, 48, 72, 96, 120, 144, 168, 192, 216 h). The results were shown in Fig. 5b in the maintext.

Thermo imaging assessments

SW8@NPs (100 μL, 1 mg mL^{-1} by tail vein injection) was administrated into nude mice bearing 143B tumors. The hyperthermia effect of **SW8@NPs** in deep-tissue under 808 nm (0.33 W cm^{-2}, 10 min) or 1064 nm (0.5 W cm^{-2}, 10 min) laser irradiation was evaluated. The thermal images and signal intensities at tumor sites were monitored at 0, 1, 2, 3, 4 mins post-injection. The results were shown in Fig. 5c and d in the maintext.

Biodistribution of SW8@NPs

The mice bearing 143B tumors were intravenously injected with 100 μL of **SW8@NPs** (1 mg mL^{-1}) through the

tail vein. Tumor tissue and major organs (heart, liver, spleen, kidney, and lung) were harvested for fluorescence imaging and biodistribution analysis at different time points after injection (0.5, 6, 24, 72, 216 h). Fecal samples were also collected every day for 18 days and used for fluorescence imaging. The results were shown in Fig. S12 and S13.

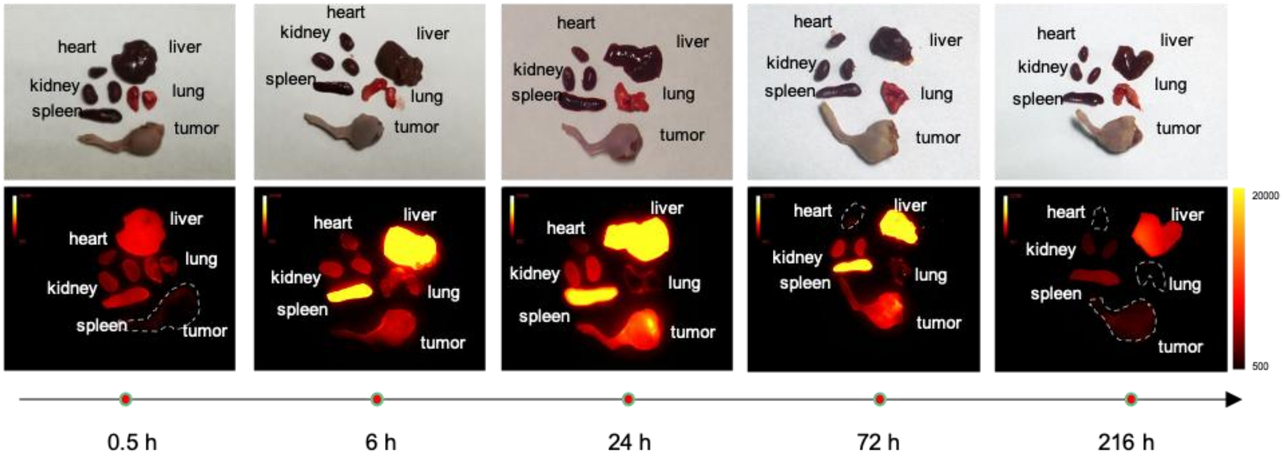

Fig. S13. Fluorescence images of vital organs isolated from mice after intravenous injection of **SW8@NPs** at designated time points.

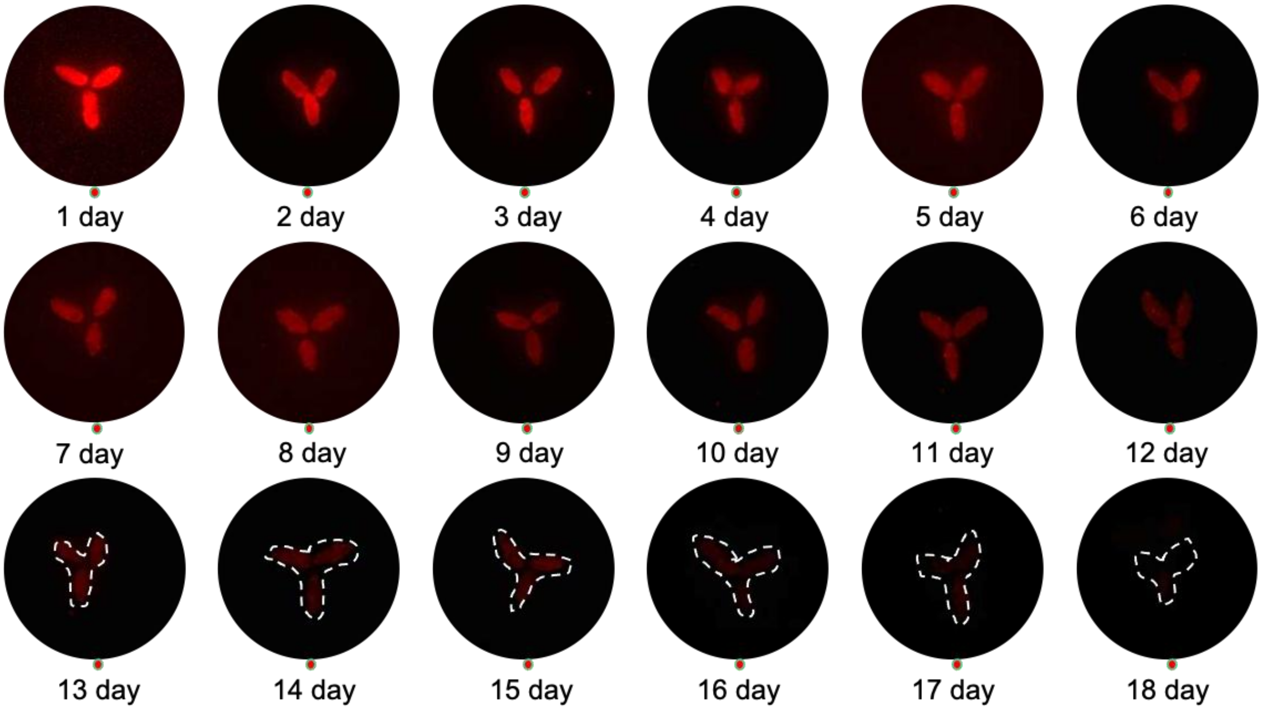

Fig. S14. Fluorescence images of the excretion behavior of **SW8@NPs** over 18 days.

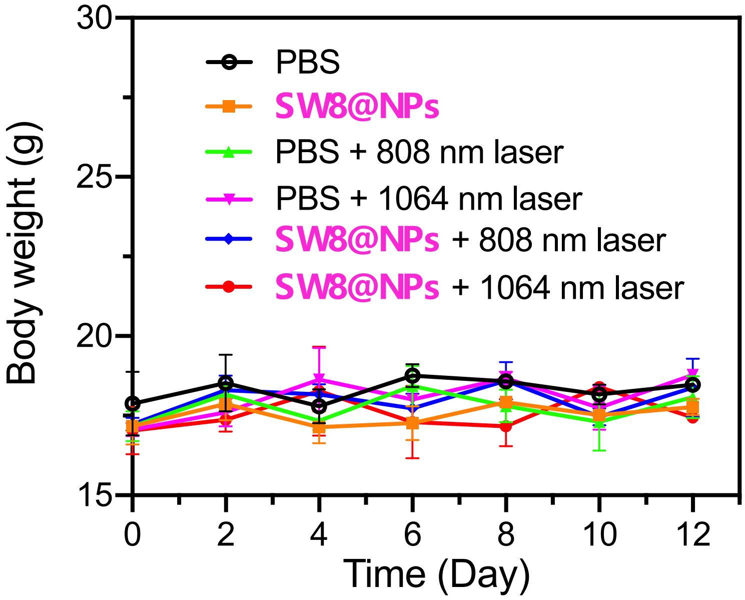

Fig. S15. Body weight changes of mice in diverse treatment groups.

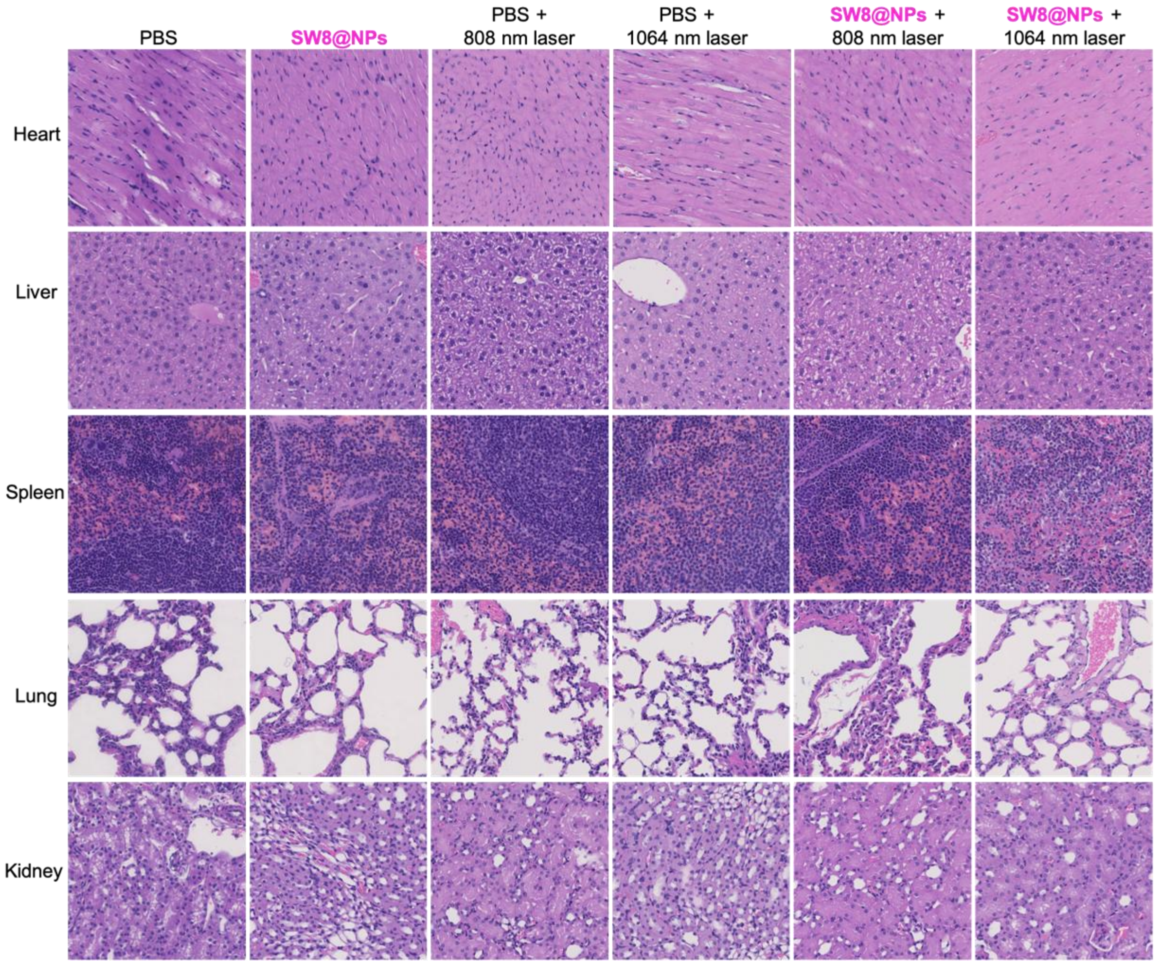

Fig. S16. Representative H&E stained images of the heart, liver, spleen, lung, and kidney, harvested from mice in the control group and treatment group.

Western blotting

Western blotting of tumor tissues was performed as described above. The results were shown in Fig. 6b in the maintext.

X-ray imaging

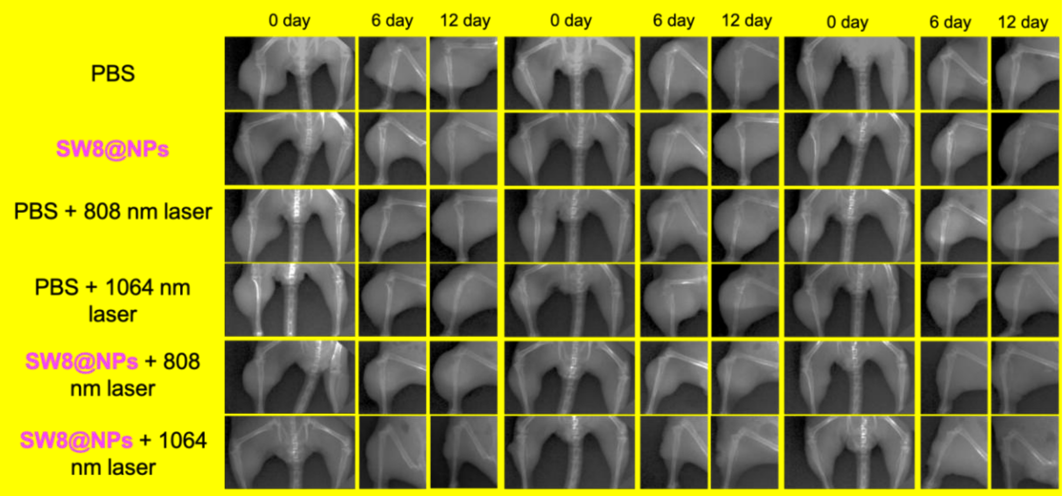

Fig. S17. The x-ray imaging in diverse treatment groups.

9. References

1. P. Prosposito, M. Casalboni, F. De Matteis, M. Glasbeek, A. Quatela, E. van Veldhoven, H. Zhang, Femtosecond dynamics of IR molecules in hybrid materials. *J. Lumin.* **94**, 641-644 (2001).

2. S. Li, Q. Deng, Y. Zhang, X. Li, G. Wen, X. Cui, Y. Wan, Y. Huang, J. Chen, Z. Liu, L. Wang, C. Lee, Rational Design of Conjugated Small Molecules for Superior Photothermal Theranostics in the NIR-II Biowindow. *Adv. Mater.* **32**, e2001146 (2020).

3. Z. Jiang, C. Zhang, X. Wang, M. Yan, Z. Ling, Y. Chen, Z. Liu, A Borondifluoride-Complex-Based Photothermal Agent with an 80% Photothermal Conversion Efficiency for Photothermal Therapy in the NIR-II Window. *Angew. Chem., Int. Ed. Engl.* **60**, 22376-22384 (2021).

4. W. Shao, Q. Wei, S. Wang, F. Li, J. Wu, J. Ren, F. Cao, H. Liao, J. Gao, M. Zhou, D. Ling, Molecular engineering of D-A-D conjugated small molecule nanoparticles for high performance NIR-II photothermal therapy. *Mater. Horiz.* **7**, 1379-1386 (2020).

5. Q. Chen, J. Chen, M. He, Y. Bai, H. Yan, N. Zeng, F. Liu, S. Wen, L. Song, Z. Sheng, C. Liu, C. Fang, Novel small molecular dye-loaded lipid nanoparticles with efficient near-infrared-II absorption for photoacoustic imaging and photothermal therapy of hepatocellular carcinoma. *Biomater. Sci.* **7**, 3165-3177 (2019).

6. H. Xiang, L. Zhao, L. Yu, H. Chen, C. Wei, Y. Chen, Y. Zhao, Self-assembled organic nanomedicine enables ultrastable photo-to-heat converting theranostics in the second near-infrared biowindow. *Nat. Commun.* **12**, 218 (2021).

10. ^{1}H- and ^{13}C-NMR Spectra.

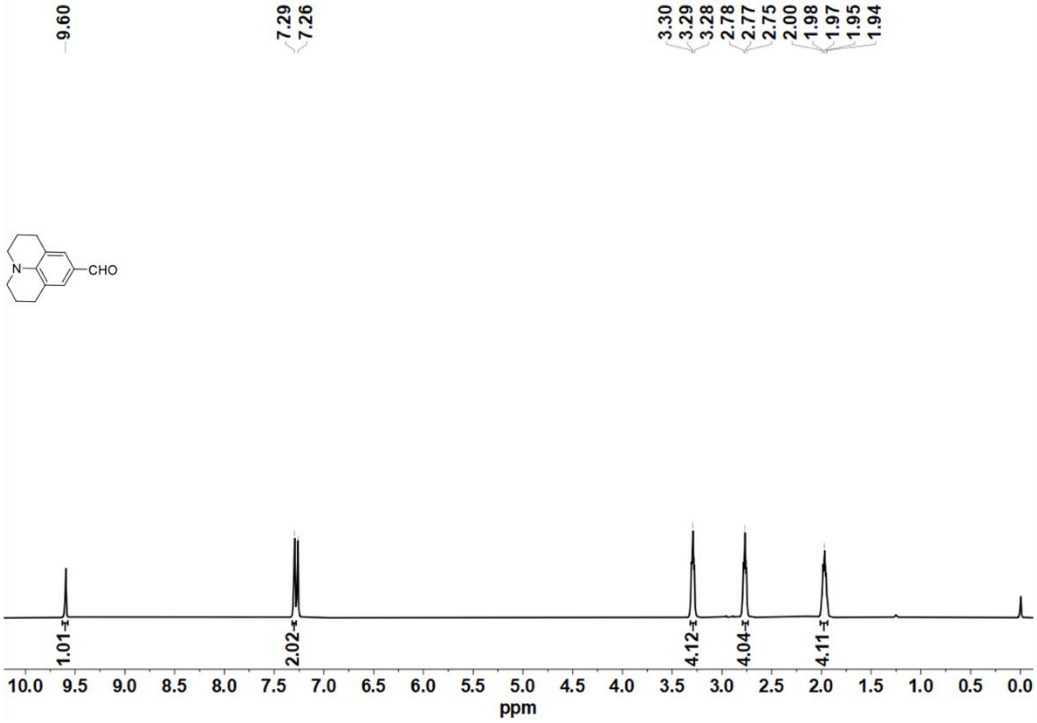

Fig. S18 ^{1}H-NMR spectra of **S1**.

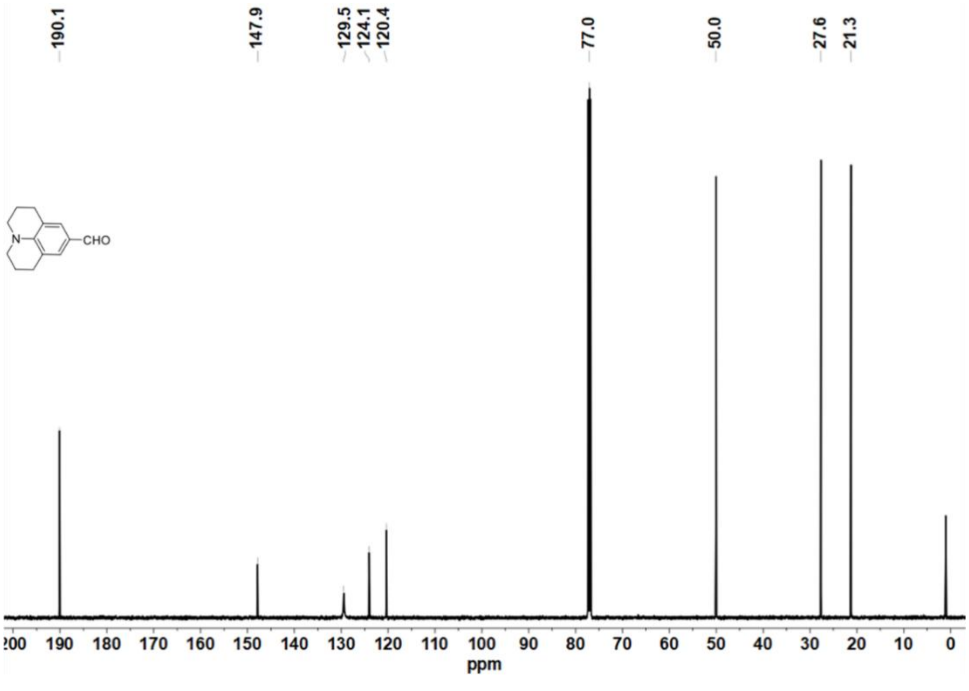

Fig. S19 ^{13}C NMR spectrum of **S1** ($CDCl_3$).

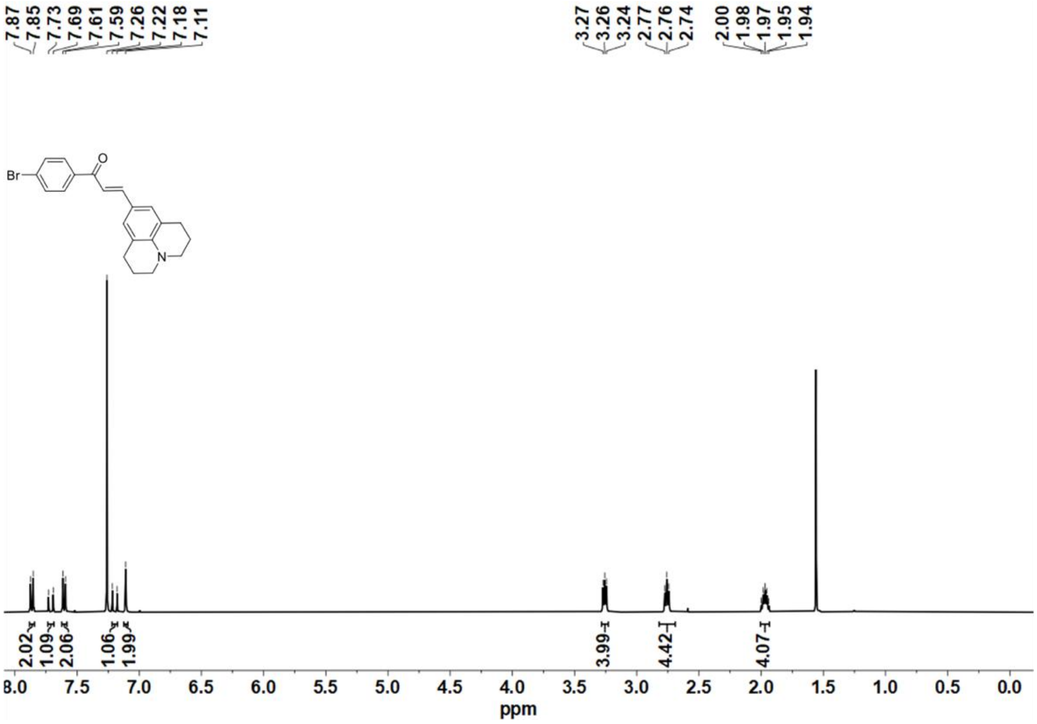

Fig. S20 ^{1}H NMR spectrum of **S2** ($CDCl_3$).

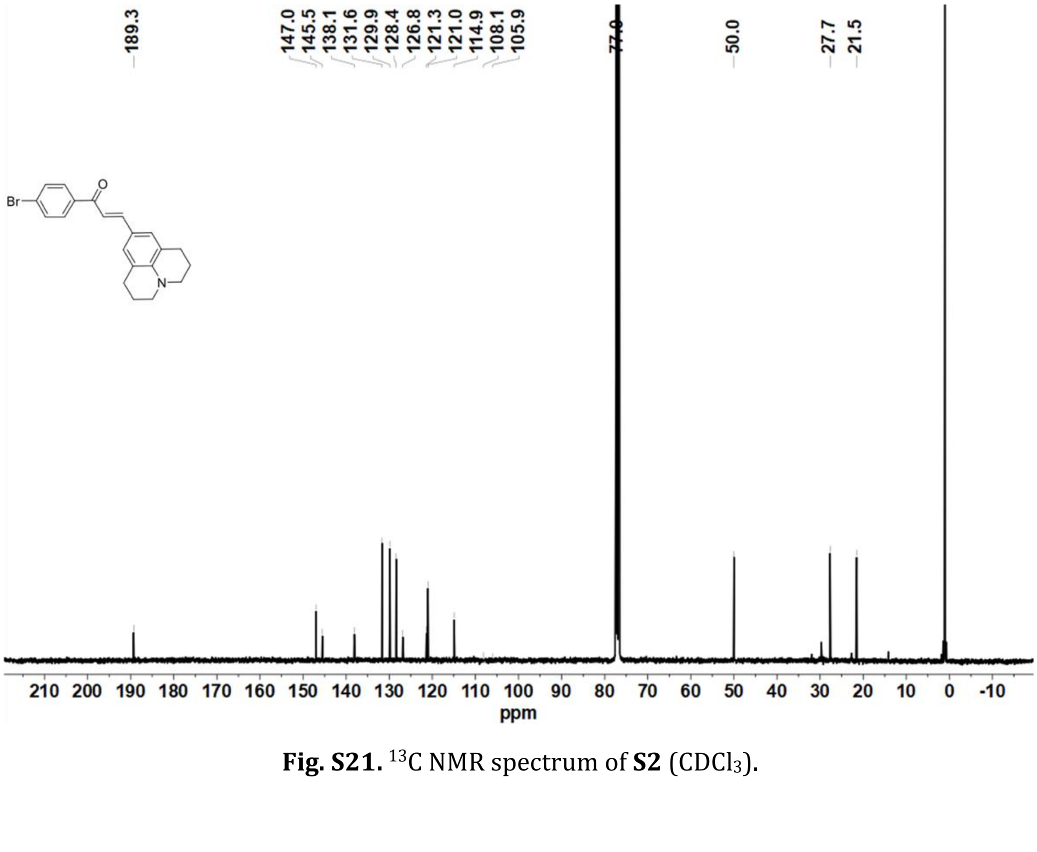

Fig. S21. ^{13}C NMR spectrum of **S2** ($CDCl_3$).

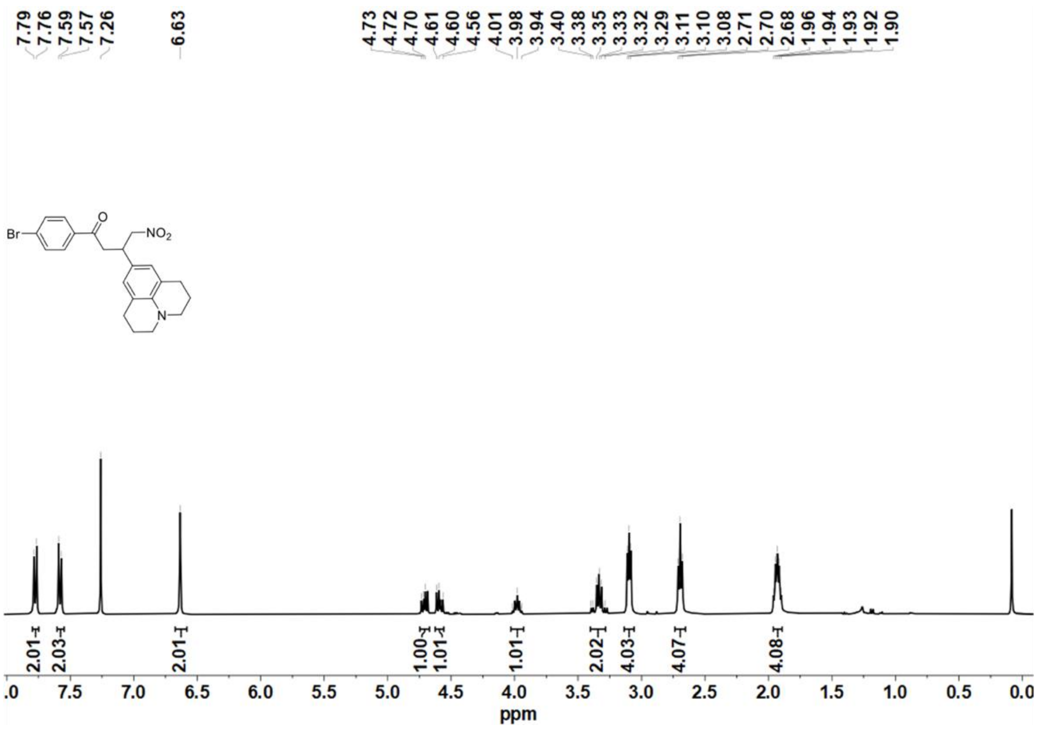

Fig. S22. ^{1}H NMR spectrum of **S3** ($CDCl_3$).

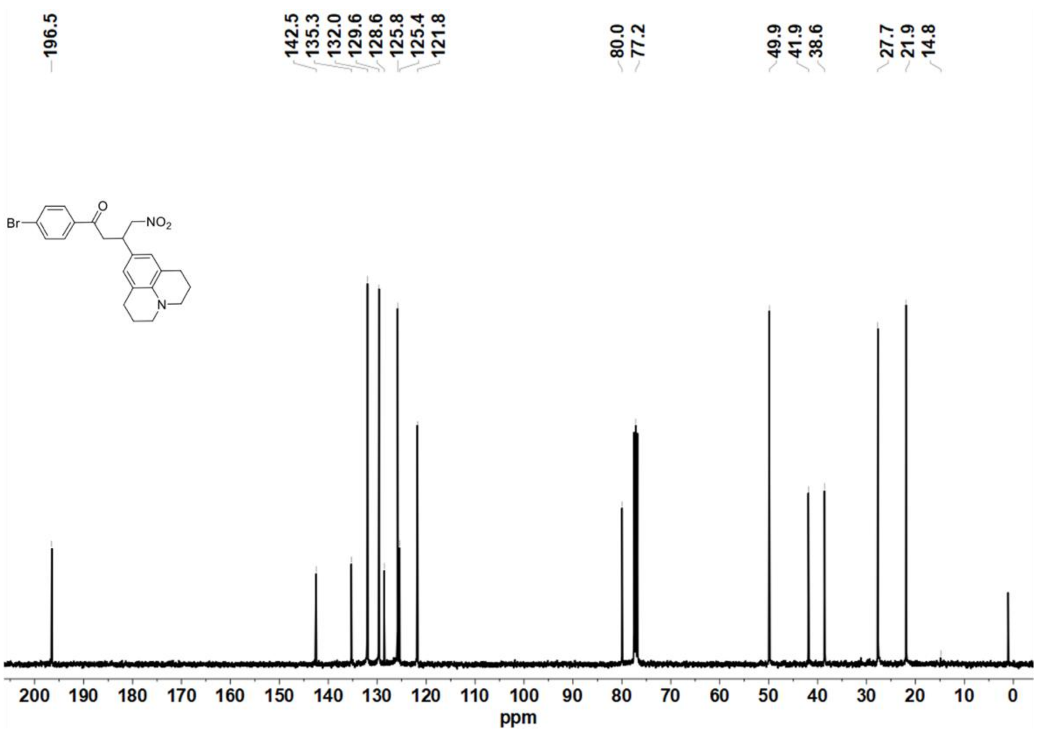

Fig. S23. ^{13}C NMR spectrum of **S3** ($CDCl_3$).

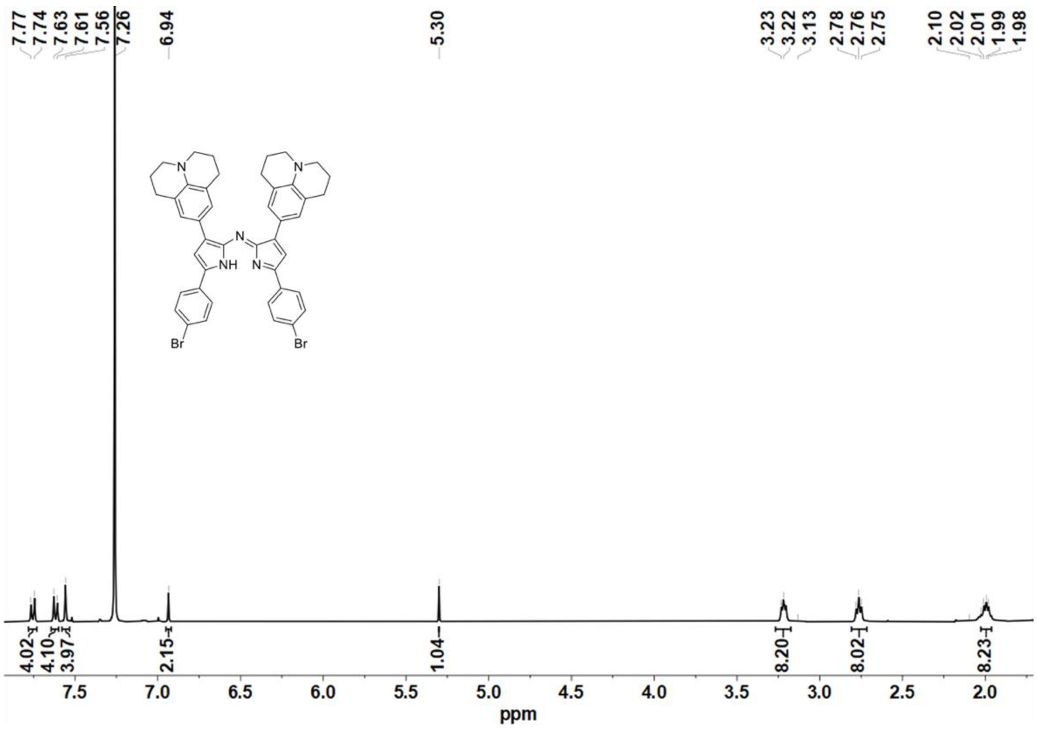

Fig. S24. ^{1}H NMR spectrum of **S4** ($CDCl_3$).

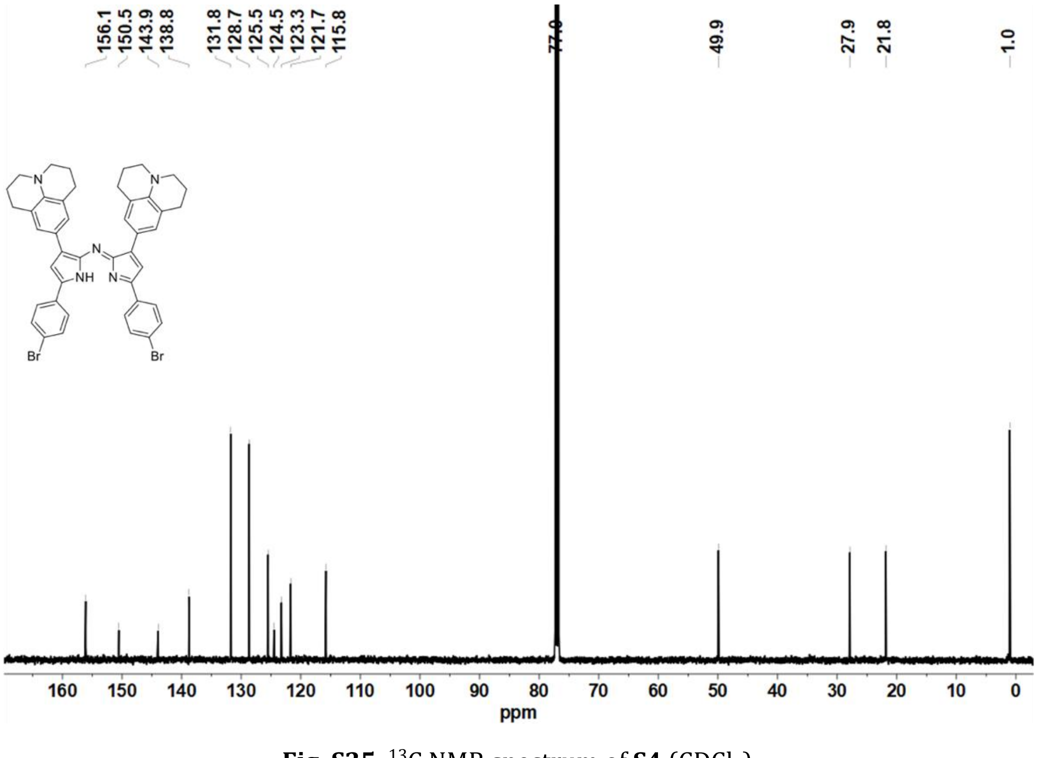

Fig. S25. ^{13}C NMR spectrum of **S4** ($CDCl_3$).

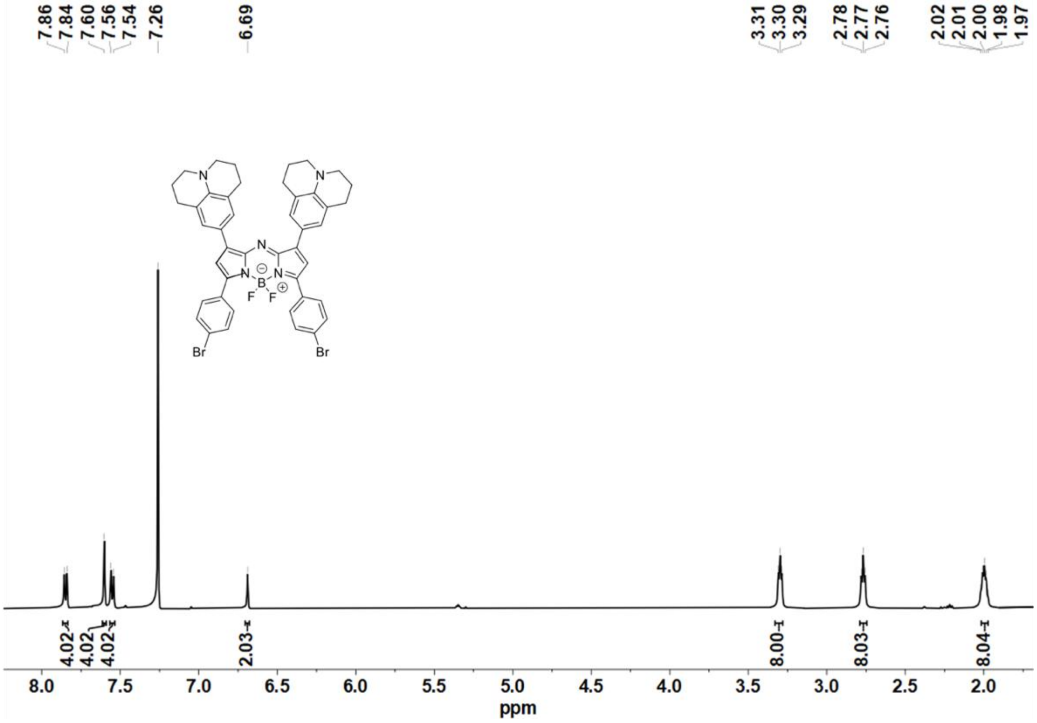

Fig. S26. ^{1}H NMR spectrum of **S5** ($CDCl_3$).

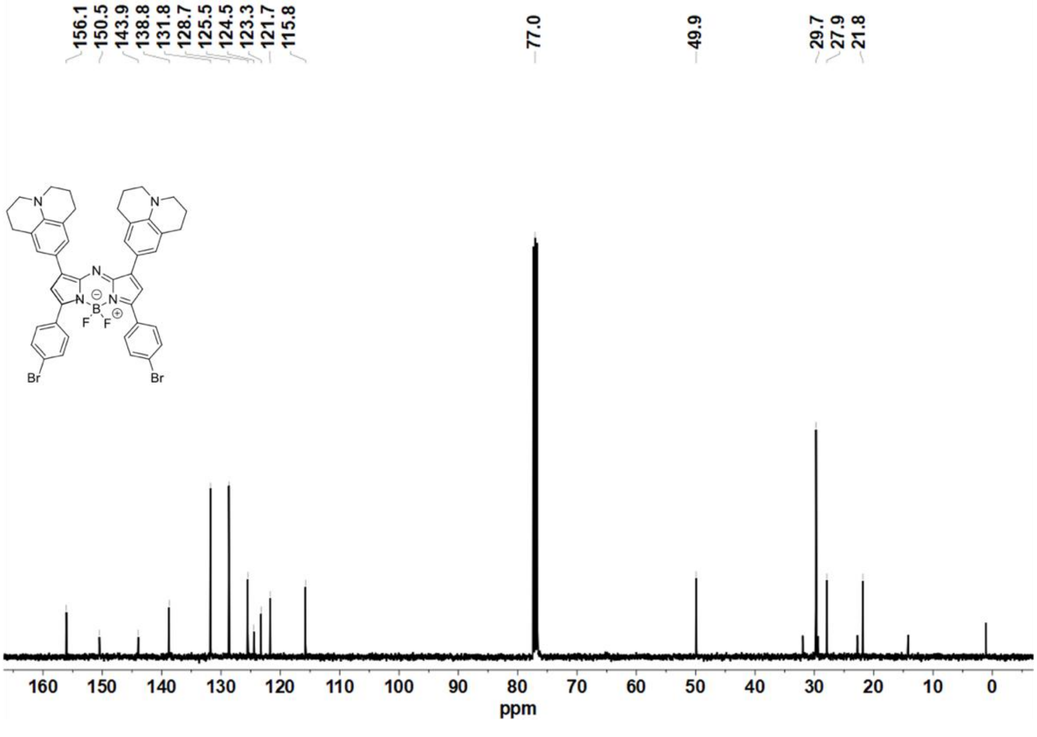

Fig. S27. ^{13}C NMR spectrum of **S5** ($CDCl_3$).

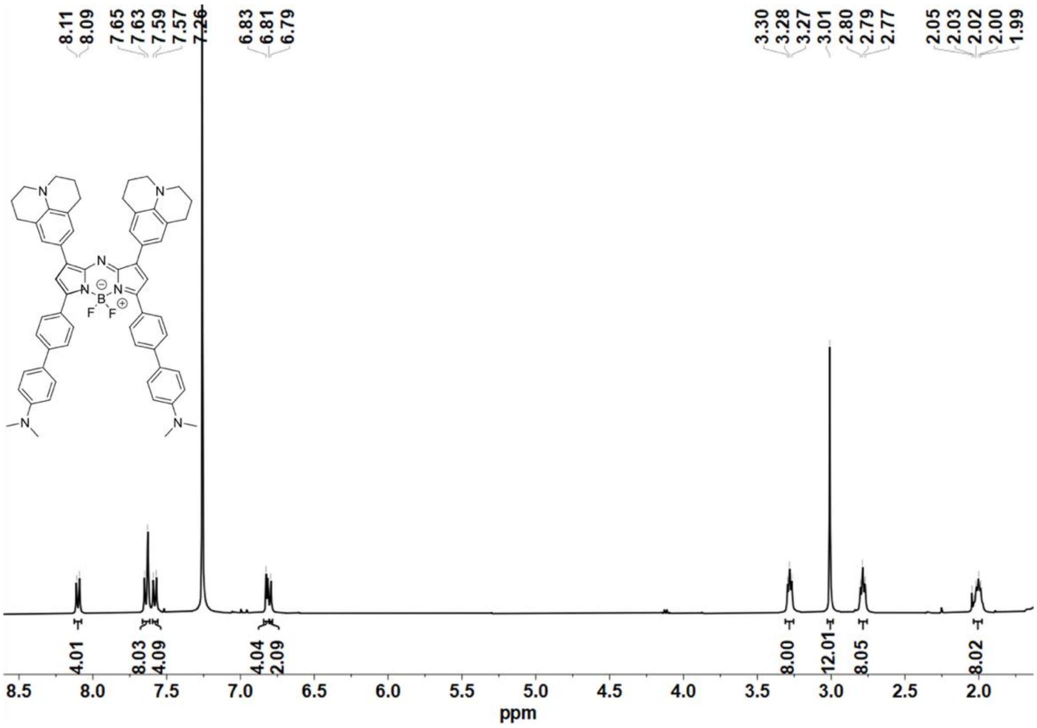

Fig. S28. ^{1}H NMR spectrum of **SW1** ($CDCl_3$).

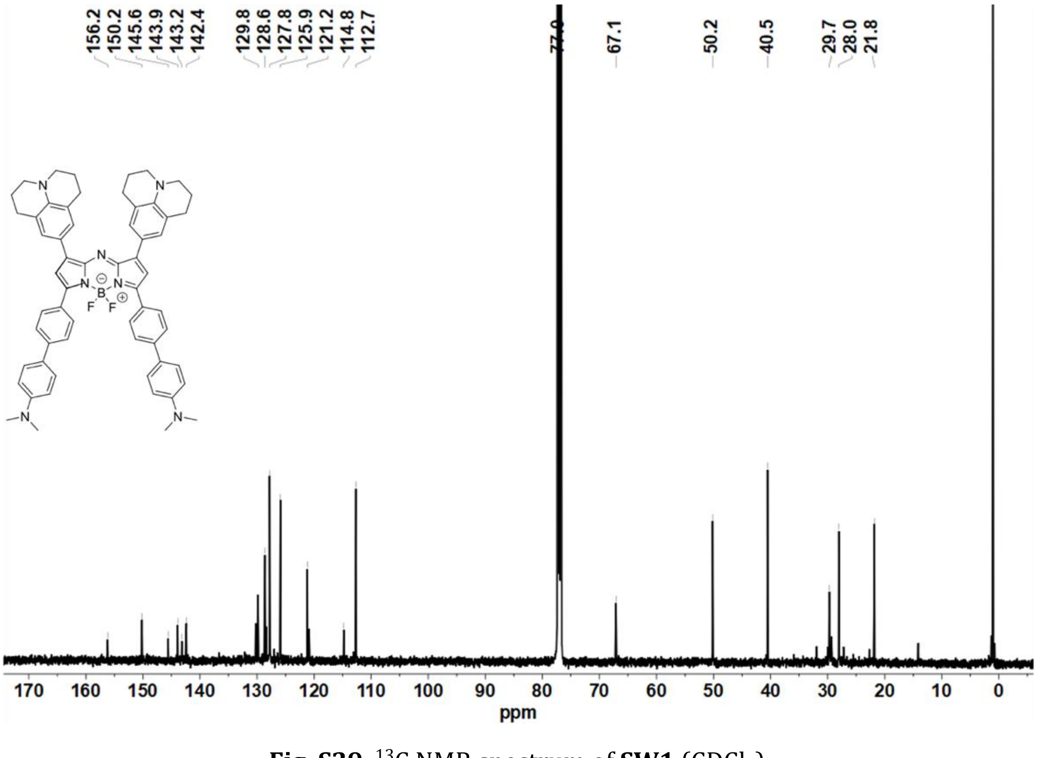

Fig. S29. ^{13}C NMR spectrum of **SW1** ($CDCl_3$).

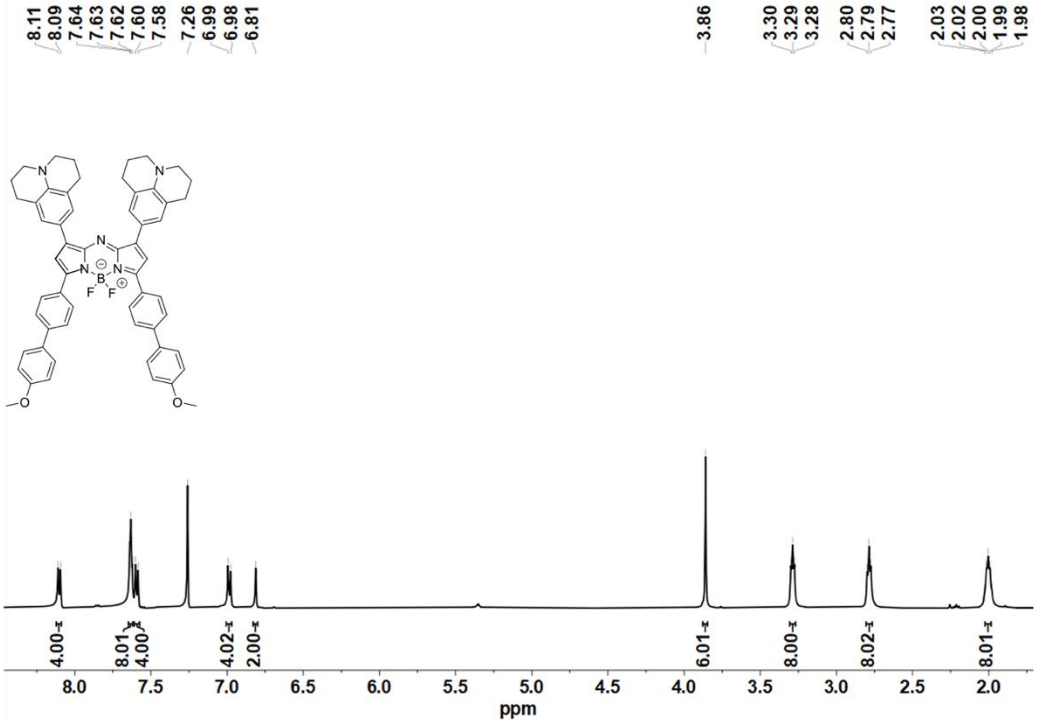

Fig. S30. ^{1}H NMR spectrum of **SW2** ($CDCl_3$).

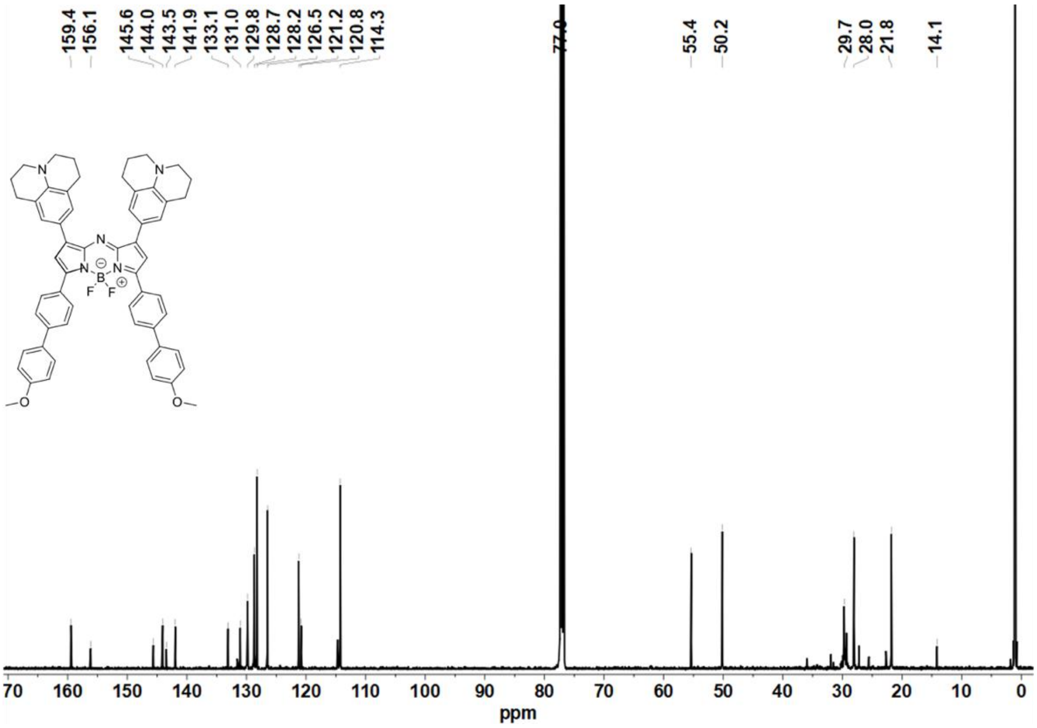

Fig. S31. ^{13}C NMR spectrum of **SW2** ($CDCl_3$).

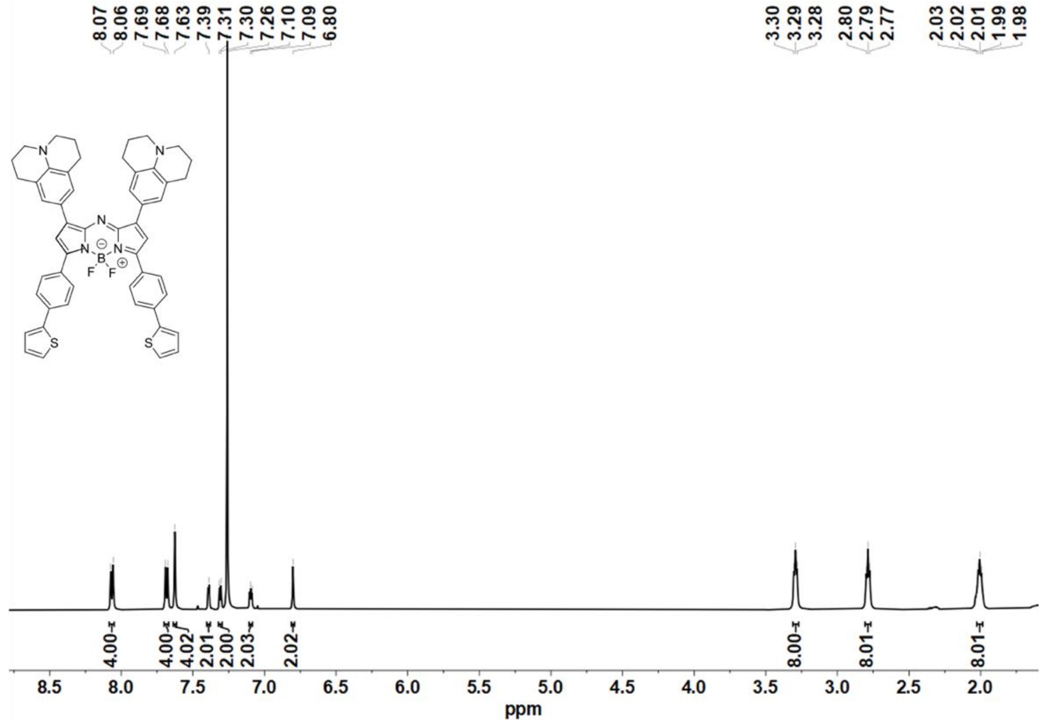

Fig. S32. ^{1}H NMR spectrum of **SW3** ($CDCl_3$).

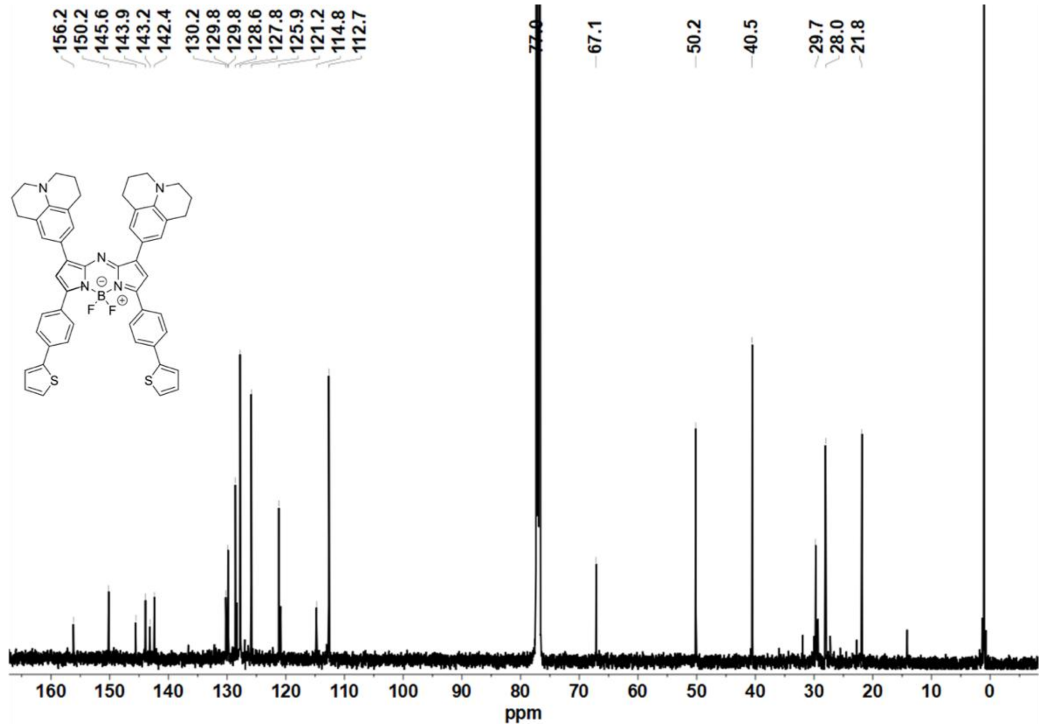

Fig. S33. ^{13}C NMR spectrum of **SW3** ($CDCl_3$).

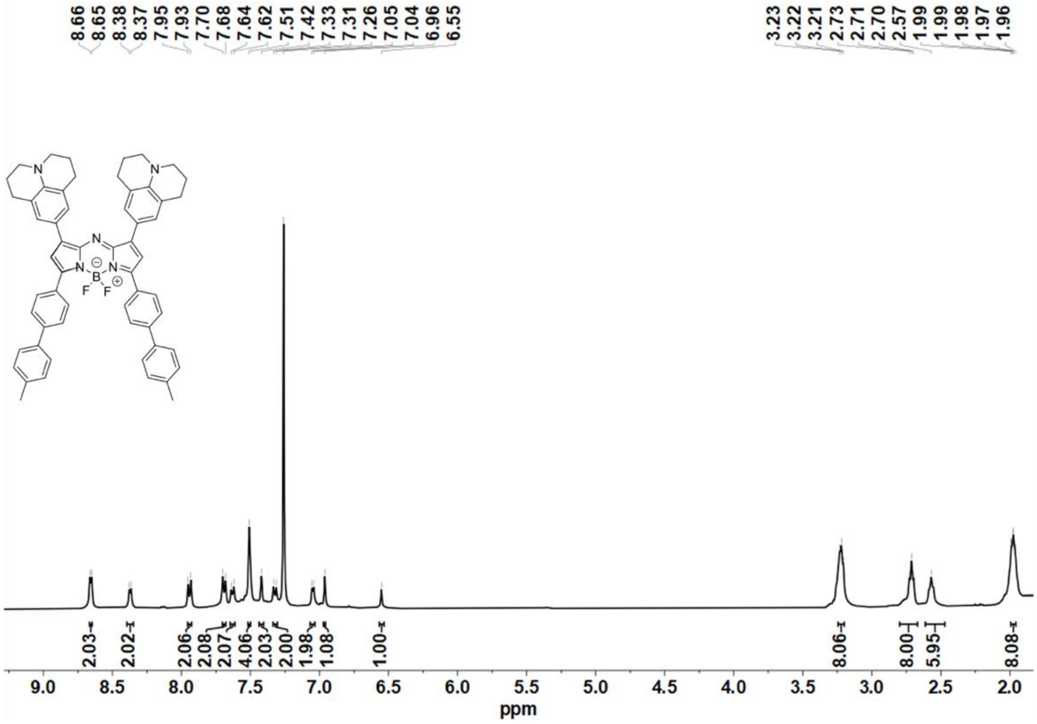

Fig. S34. ^{1}H NMR spectrum of **SW4** ($CDCl_3$).

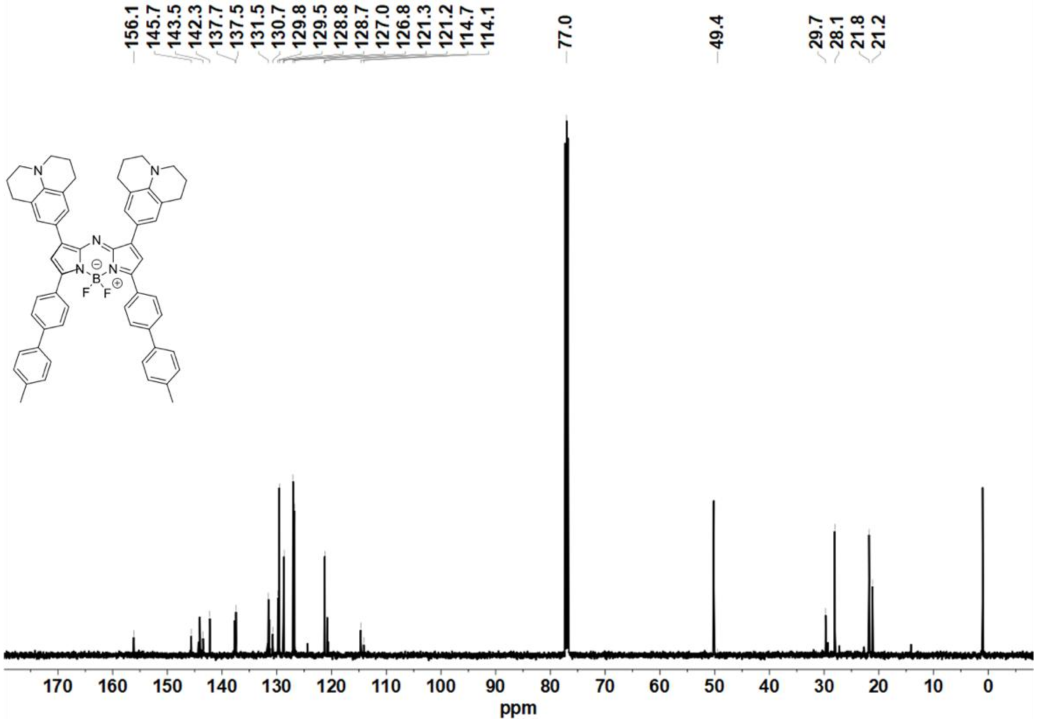

Fig. S35. ^{13}C NMR spectrum of **SW4** ($CDCl_3$).

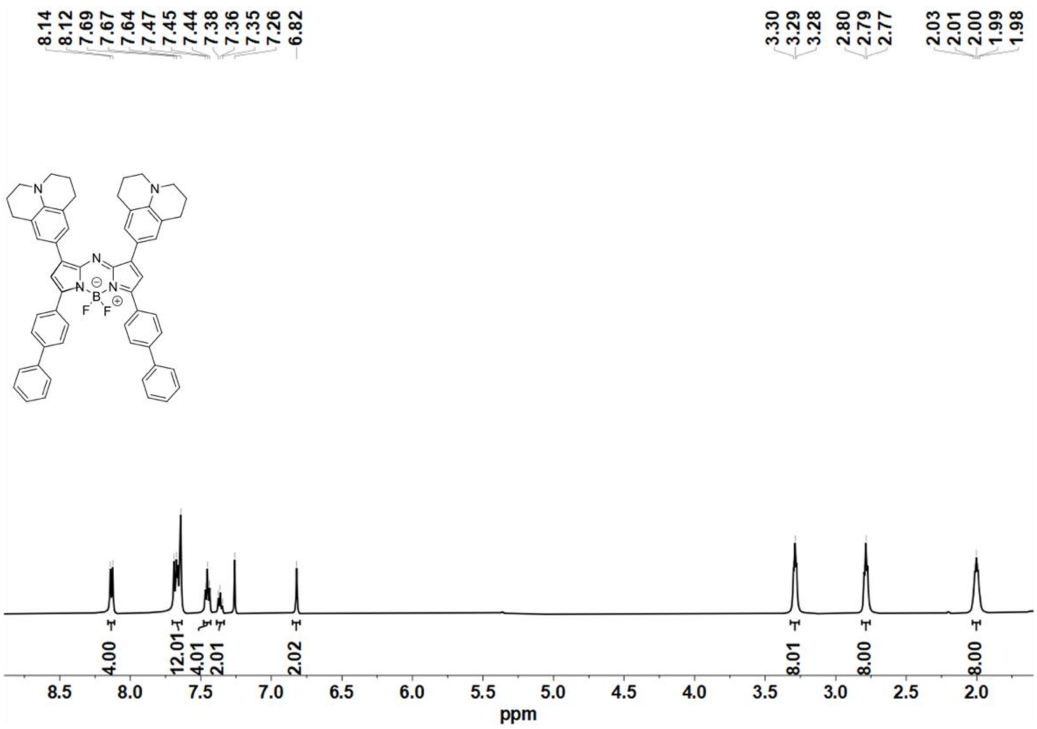

Fig. S36. ^{1}H NMR spectrum of **SW5** ($CDCl_3$).

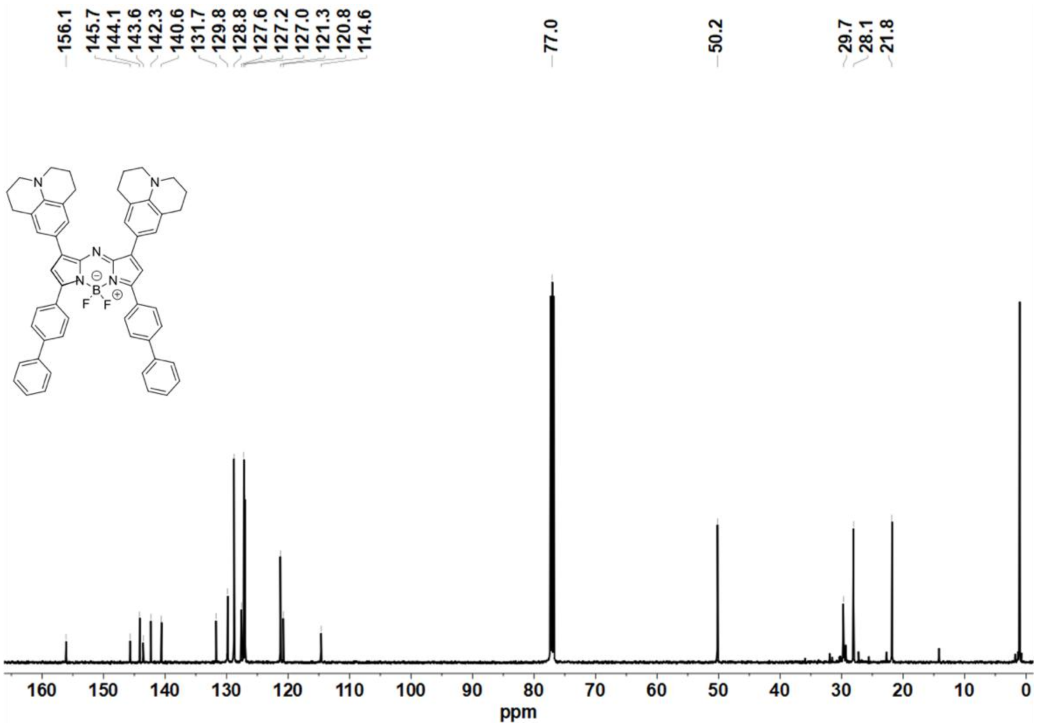

Fig. S37. ^{13}C NMR spectrum of **SW5** ($CDCl_3$).

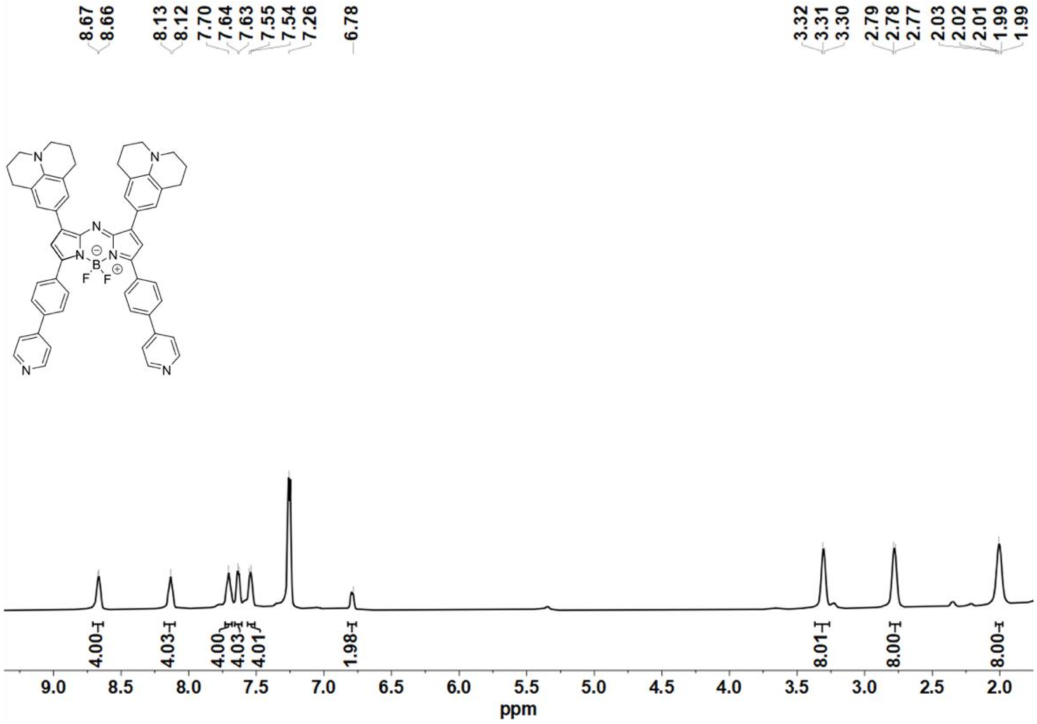

Fig. S38. ^{1}H NMR spectrum of **SW6** ($CDCl_3$).

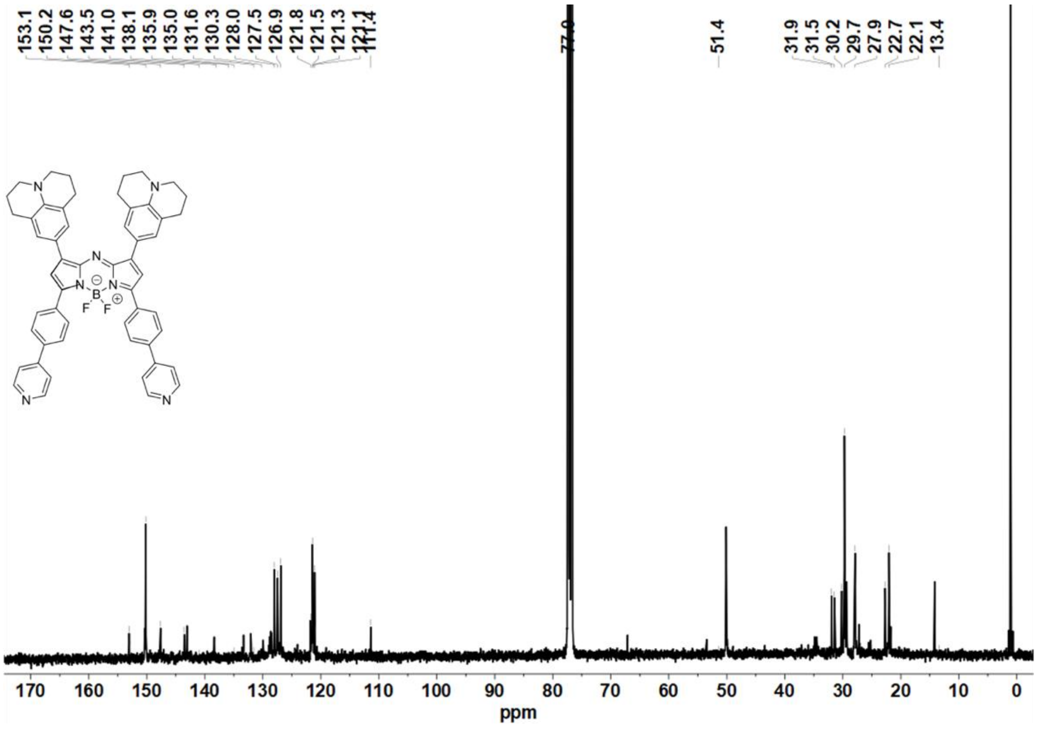

Fig. S39. ^{13}C NMR spectrum of **SW6** ($CDCl_3$).

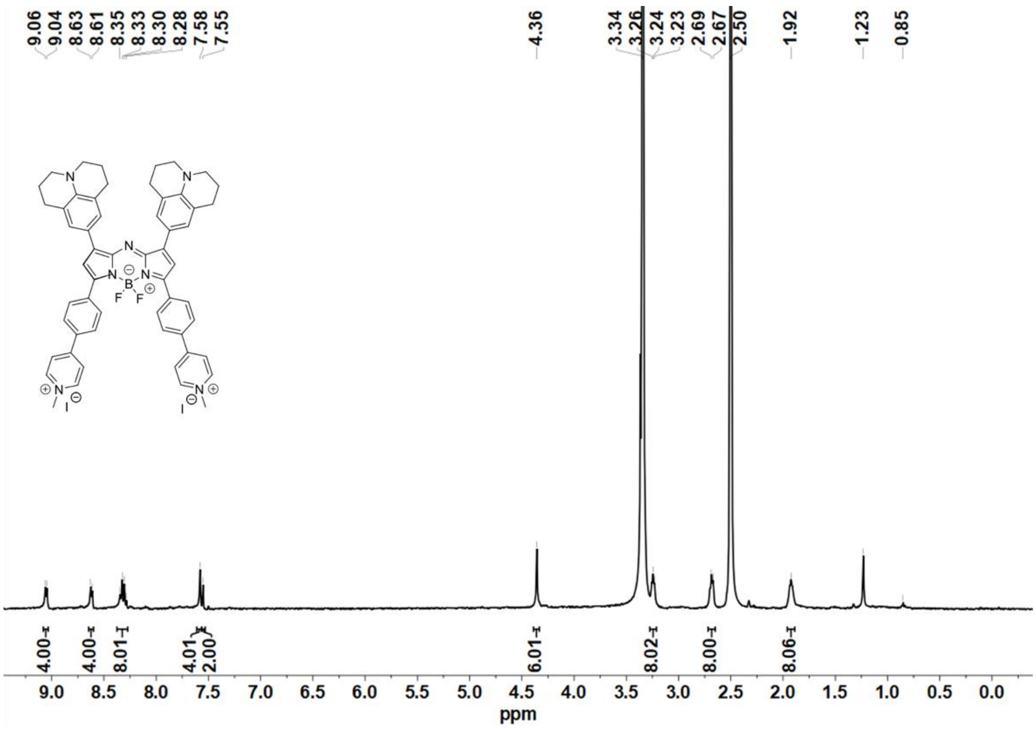

Fig. S40. ^{1}H NMR spectrum of **SW7** (d_6-DMSO).

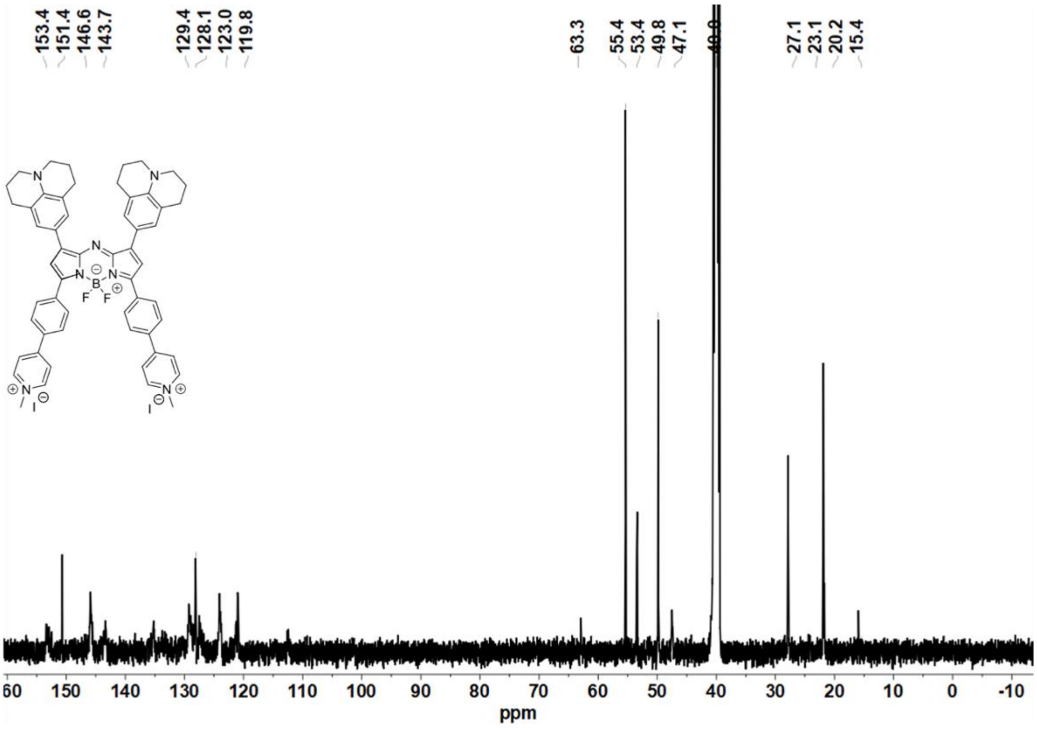

Fig. S41. ^{13}C NMR spectrum of **SW7** (d_6-DMSO).

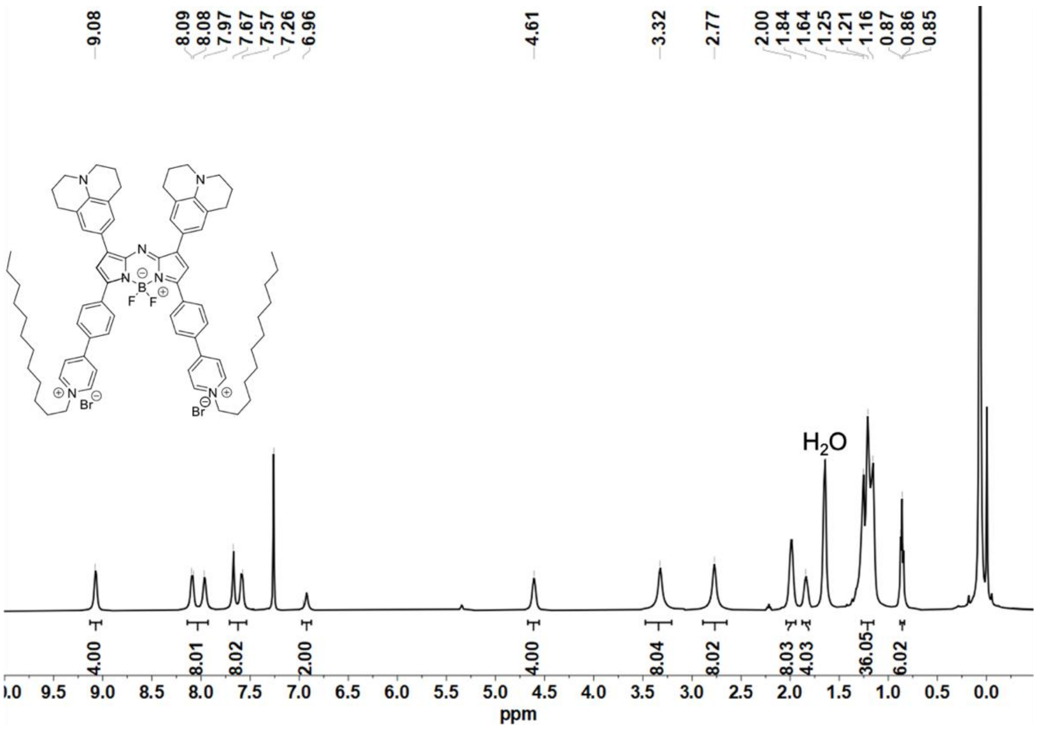

Fig. S42. ^1H NMR spectrum of **SW8** ($CDCl_3$).

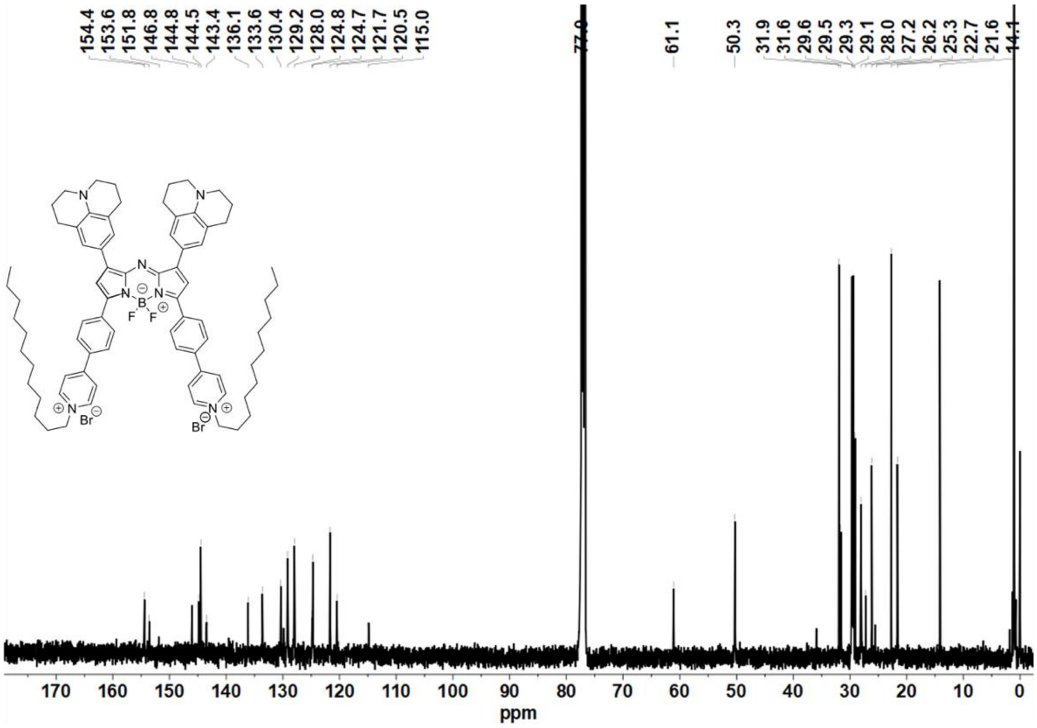

Fig. S43. ^{13}C NMR spectrum of **SW8** ($CDCl_3$).
